# Palladium-based nanomaterials for cancer imaging and therapy

**DOI:** 10.7150/thno.45990

**Published:** 2020-08-08

**Authors:** Yongchun Liu, Jingchao Li, Mei Chen, Xiaolan Chen, Nanfeng Zheng

**Affiliations:** 1 College of Materials Science and Engineering, Hunan University, Changsha, China.; 2 Department of Chemistry, Xiamen University, Xiamen, China.

**Keywords:** Palladium-based nanomaterials, cancer imaging, photothermal therapy, combined cancer therapy, safety profile

## Abstract

In recent decade, palladium-based (Pd-based) nanomaterials have shown significant potential for biomedical applications because of their unique optical properties, excellent biocompatibility and high stability in physiological environment. Compared with other intensively studied noble nanomaterials, such as gold (Au) and silver (Ag) nanomaterials, research on Pd-based nanomaterials started late, but the distinctive features, such as high photothermal conversion efficiency and high photothermal stability, have made them getting great attention in the field of nanomedicine. The goal of this review is to provide a comprehensive and critical perspective on the recent progress of Pd-based nanomaterials as imaging contrast agents and therapeutic agents. The imaging section focuses on applications in photoacoustic (PA) imaging, single-photon emission computed tomography (SPECT) imaging, computed tomography (CT) imaging and magnetic resonance (MR) imaging. For treatment of cancer, single photothermal therapy (PTT) and PTT combined with other therapeutic modalities will be discussed. Finally, the safety concerns, forthcoming challenges and perspective of Pd-based nanomaterials on biomedical applications will be presented.

## Introduction

Cancer has become one of the world's most significant health threats, because of its high mortality and morbidity 1. Although great effort has been devoted to the battle against cancer, effective treatment remains a huge challenge owing to the complicated heterogeneity and metastasis pathways of malignant tumors. Hence, it is imperative to explore more effective diagnostic and treatment strategies to overcome the limitations of conventional therapies. The past two decades have witnessed a rapid growth in the wide application of nanomaterials in biomedicine, including biosensing, molecular imaging, drug delivery and cancer therapy 2, 3. The shapes and sizes of nanomaterials are controllable and their surfaces can be diversely modified, thus their related properties can be regulated correspondingly 4. Combinations of their inherent properties with other functional components, i.e., molecular probes, chemotherapy drugs and photosensitizers, can contribute to the construction of multifunctional cancer theranostic nanoplatforms 5.

Palladium-based (Pd-based) nanomaterials, such as Pd nanosheets (NSs), porous/hollow Pd nanoparticles (NPs) and Pd@M (M=Ag, Au, Pt, SiO_2_, ZIF-8) nanocomposites, exhibit strong absorption in the near-infrared (NIR) region, as well as high photothermal conversion efficiency, excellent photothermal stability, and good biocompatibility. Taking advantage of these distinctive features, Pd-based nanomaterials have become prominent candidates as cancer imaging contrast agents and therapeutic agents. To date, the synthesis and properties of Pd-based nanomaterials have been discussed in several review papers 6-8. Herein, we will give a brief introduction to the typical Pd-based nanomaterials in cancer theranostics. Next, the discussion focuses on the recent advances in cancer imaging, including photoacoustic (PA) imaging, single-photon emission computed tomography (SPECT) imaging, computed tomography (CT) imaging and magnetic resonance (MR) imaging. For cancer therapy, such as single photothermal therapy (PTT) and PTT combined with other therapy modalities will also be discussed. In addition, the application of Pd-based nanomaterials in prodrug activation will be highlighted. The safety profiles and future challenges of Pd-based nanomaterials will also be included. We hope this review will provide comprehensive and deep insight for Pd-based nanomaterials in biomedicine and open new possibilities for future clinical applications.

## Palladium-based nanomaterials

**Figure 1:**
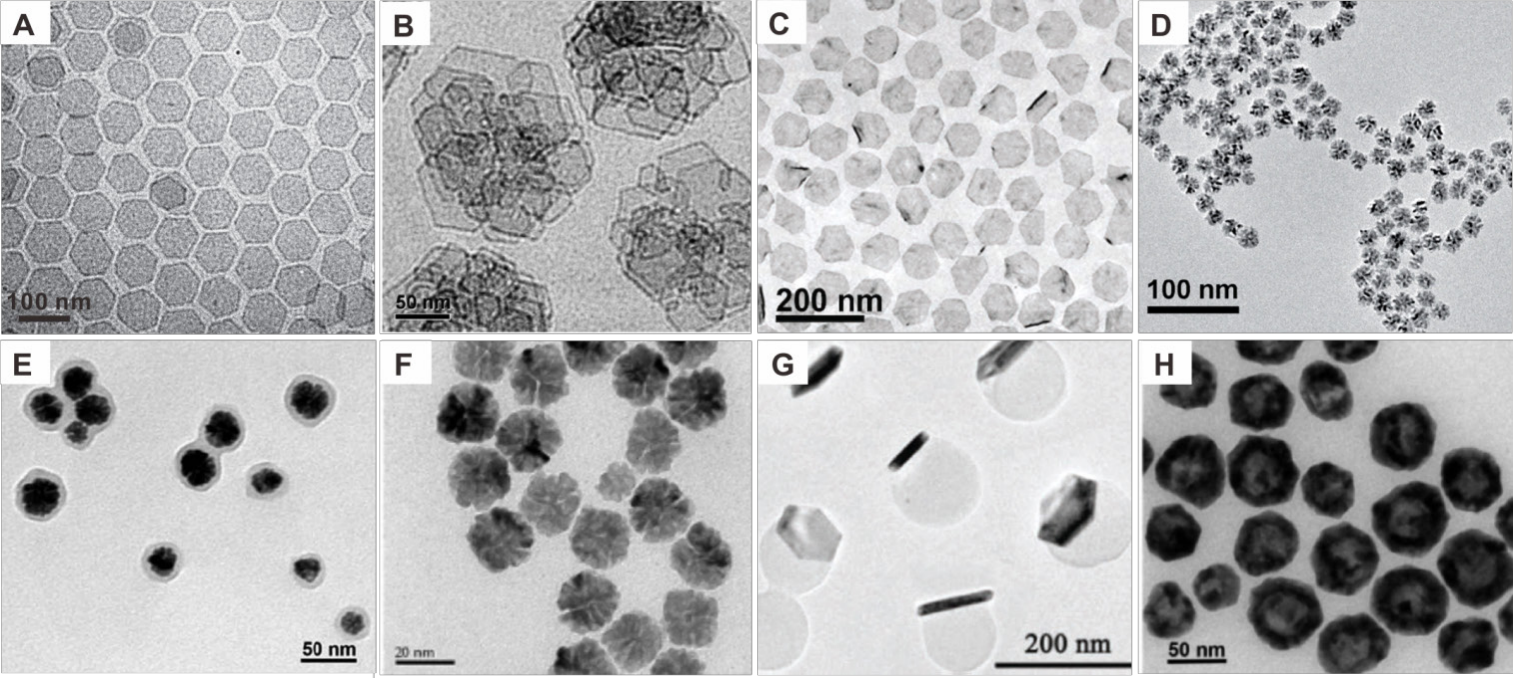
TEM images of (**A**) Pd NSs, (**B**) Pd collora, (**C**) Pd@Ag nanoplates, (**D**) Porous Pd NPs, (**E**) Pd NPs@PPy, (**F**) Pd NPs, (**G**) PdNS/ZIF-8 JNPs and (**H**) H-Pd nanospheres. Adapted with permission from ref. 10, Copyright from 2011, Springer Nature; adapted with permission from ref. 13. Copyright 2011, American Chemical Society; adapted with permission from ref. 14. Copyright 2011, Wiley-VCH; adapted with permission from ref. 18. Copyright 2014, Royal Society of Chemistry; adapted with permission from ref. 19. Copyright 2016, Royal Society of Chemistry; adapted with permission from ref. 20. Copyright 2018, Springer Nature; adapted with permission from ref. 21. Copyright 2018, Wiley-VCH; adapted with permission from ref. 22. Copyright 2018, American Chemical Society.

Compared with other intensively studied noble nanomaterials, i.e., Au and Ag nanostructures 9, research on biomedical application of Pd-based nanomaterials started late, mainly because the optical absorption of Pd NPs is principally located in the UV-Vis region, resulting in less favorable attention. Significant advances of Pd-based nanomaterials in biomedical fields began with the successful synthesis of ultrathin two-dimensional (2D) Pd NSs in 2011 (Figure 1A) 10. As typical 2D nanomaterials, Pd NSs exhibit strong NIR absorption, high photothermal conversion efficiency, excellent photothermal stability and good biocompatibility. The sizes of Pd NSs could be easily tuned from 5 nm to 120 nm, while adapting uniform hexagonal morphology 10, 11.The optical absorption peaks of Pd NSs vary with size but are mostly located in the NIR region, making Pd NSs promising candidates for PTT. Since then, the biomedical application of Pd-based nanomaterials has attracted increasing interests 8, 12. To further improve the stability, intracellular uptake efficiency and tumor targeting ability, Pd collora (Figure 1B) 13, Pd@Ag (Figure 1C) 14, Pd@Au 15, Pd@Pt 16, and Pd@SiO_2_ 17 were developed. In addition to 2D nanomaterials, Pd-based nanomaterials with various shapes and functions have been explored by many groups, such as porous Pd NPs (Figure 1D) 18, Pd NPs@PPy (Figure 1E) 19, Pd NPs linked with RGD peptide (Figure 1F) 20, Pd NS/ZIF-8 with Janus nanostructure (Figure 1G) 21 and hollow Pd nanospheres (Figure 1H) 22. Besides the strong NIR absorption, Pd-based nanomaterials can be integrated with different imaging and therapeutic modalities to form multifunctional nanoplatforms. The application of Pd-based nanomaterials as imaging contrast agents in PA imaging, SPECT imaging, CT imaging and MR imaging are summarized in Table 1. Cancer therapies based on Pd-based nanomaterials in single PTT and PTT combined with other therapy modalities are summarized in Table 2.

## Bioimaging applications of Pd-based nanomaterials

With the rapid progress in imaging technologies, development of effective contrast agents has attracted increasing attentions. Herein, a series of bioimaging applications based on Pd nanomaterials will be discussed, including PA imaging, CT imaging, SPECT imaging and MR imaging. From the view of the imaging mechanisms, the PA and CT imaging can be realized by Pd-based nanomaterials themselves because of their inherent properties, while SPECT and MR imaging rely on integrated contrast agents, such as ^125^I and Gd-DTPA, which can be loaded on Pd-based nanocarriers and delivered to tumor sites. All of these imaging modalities show unique advantages and can complement each other to achieve accurate diagnosis with maximum sensitivity and specificity.

### PA imaging

Photoacoustic (PA) imaging has shown great promise as a novel biomedical imaging modality that combines the high contrast of optical imaging with the high spatial resolution of ultrasound 23, 24. The imaging signal is generated by the acoustic waves induced through the absorption of a pulsed laser beam by specific tissues, and can provide noninvasive images with high contrast and spatiotemporal resolution. Different from optical imaging, the acoustic signal of PA imaging exhibits positive correlation with the light absorption capacity of imaged tissues and can image deeper tissues. In order to reduce the non-specific absorption and optimize the PA imaging contrast, resolution and penetration depth, contrast agents with high optical absorption coefficient and photothermal conversion efficiency were introduced to improve the signal-noise ratio by altering the contrast between the imaged tissue and surrounding tissue. Besides endogenous chromophores, such as melanin and hemoglobin, much effort has been devoted to the development of exogenous contrast agent with high stability and high optical absorption 25, 26. Among them, nanomaterials, especially those with high optical absorption coefficients in the NIR region, excellent photostability upon laser irradiation and good biocompatibility, have drawn extensive attention as PA imaging contrast agents 27-30.

**Figure 2:**
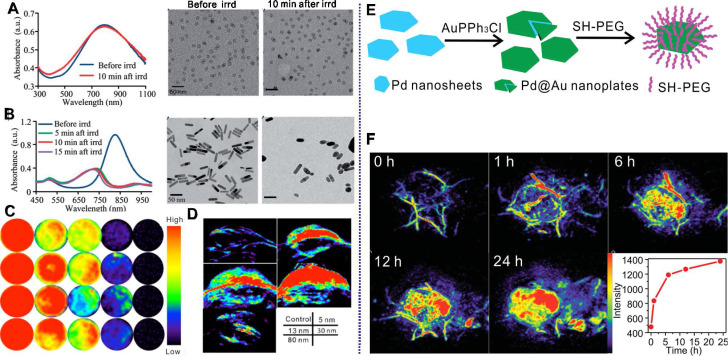
Optical spectrum and TEM images of (**A**) Pd NSs and (**B**) Au nanorods before and after laser irradiation. PA imaging application of different sized Pd NSs (**C**) *in vitro* and (**D**) *in vivo*. (**E**) Synthetic procedure for Pd@Au nanoplates and (**F**) their application in PA imaging of tumor. Adapted with permission from ref. 31. Copyright 2014, Royal Society of Chemistry; adapted with permission from ref. 11, Copyright from 2017, Springer Nature; adapted with permission from ref. 15. Copyright 2014, Wiley-VCH.

As mentioned above, the successful synthesis of Pd NSs paves the way for the application of Pd nanomaterials in PA imaging. Pd NSs exhibit strong absorption in the NIR region, thus holding great potential as PA imaging contrast agents. Nie et al. first demonstrated the application of Pd NSs in cancer PA imaging 31. Pd NSs, with diameter of 16 nm, have strong NIR absorption and allow enhanced cancer PA imaging after intravenous (i.v.) injection in tumor-bearing mice. Different from Au nanorods, Pd NSs exhibit high stability and are able to maintain their morphology and absorption after long-term laser irradiation (Figure 2A-B). Pd NSs with sizes ranging from 5 nm to 80 nm exhibited excellent PA imaging *in vitro*, while superior contrast effect can be observed in mice injected with smaller sized Pd NSs (5 nm, 13 nm and 30 nm) due to the size dependent accumulation behavior *in vivo* (Figure 2C-D) 11. Utilizing 30 nm Pd NSs as seeds, Chen et al. reported the fabrication of Pd@Au nanoplates via a seeded regrowth method. PEGylated Pd@Au nanoplates showed strong absorption for NIR light, excellent photostability and ultra-high tumor accumulation efficiency (Figure 2E) 15. Thus, PEGylated Pd@Au nanoplates have been successfully applied for real-time monitoring the accumulation in tumor sites and PA imaging guided cancer therapy (Figure 2F). Recently, Yang et al. synthesized novel Pd@Au nanoplates with partial coating of Au on Pd NSs and explored their potential application as PA imaging contrast agents 32. Zhao and coworkers developed Pd hydride nanomaterials (PdH_0.2_) through the incorporation of hydrogen into the lattice of Pd nanocubes 33. Compared with Pd nanocubes, the absorption of PdH_0.2_ nanocubes in the NIR region was obviously enhanced; therefore PdH_0.2_ nanocubes were successfully applied in PA imaging-guided hydrogenothermal cancer therapy.

### SPECT imaging

Single photon emission computed tomography (SPECT), as a quantitative nuclear imaging method, can provide 3-dimensional spatial images of the *in vivo* distribution of injected radioisotopes, and plays an important role in preclinical and clinical research 34-36. The commonly used radioisotopes for SPECT include ^99m^Tc 37, ^131^I 38, ^125^I 39, ^111^In 40, ^177^Lu 41, and ^67^Ga 42. Compared with traditional optical imaging technologies, SPECT exhibits great potential in clinical applications with unlimited penetration depth. Recently, nanomaterials have been applied as nanocarriers to regulate the behavior of radioisotopes *in vivo*, and the pharmacokinetics and tumor uptake of nanomaterials can also be presented in a non-invasive manner through SPECT imaging. Chelating moieties are usually required for the stable labelling of radioisotopes by the formation of coordination complexes on the surface of nanomaterials 43. Zhong et al. successfully radiolabeled polydopamine (PDA) nanoparticles with ^99m^Tc for SPECT imaging through the chelation reaction of ^99m^TcO_4_^-^ solution with the functional groups on PDA and performed nuclear-imaging-guided cancer therapy 44. For the radiolabeling of ^131^I on the surface of nanomaterials, electrophilic substitution reactions followed by oxidation of iodide ions in the presence of chloramine-T. Song et al. developed ^131^I-labeled porous hollow Pd NPs utilizing a similar procedure 45.

**Figure 3:**
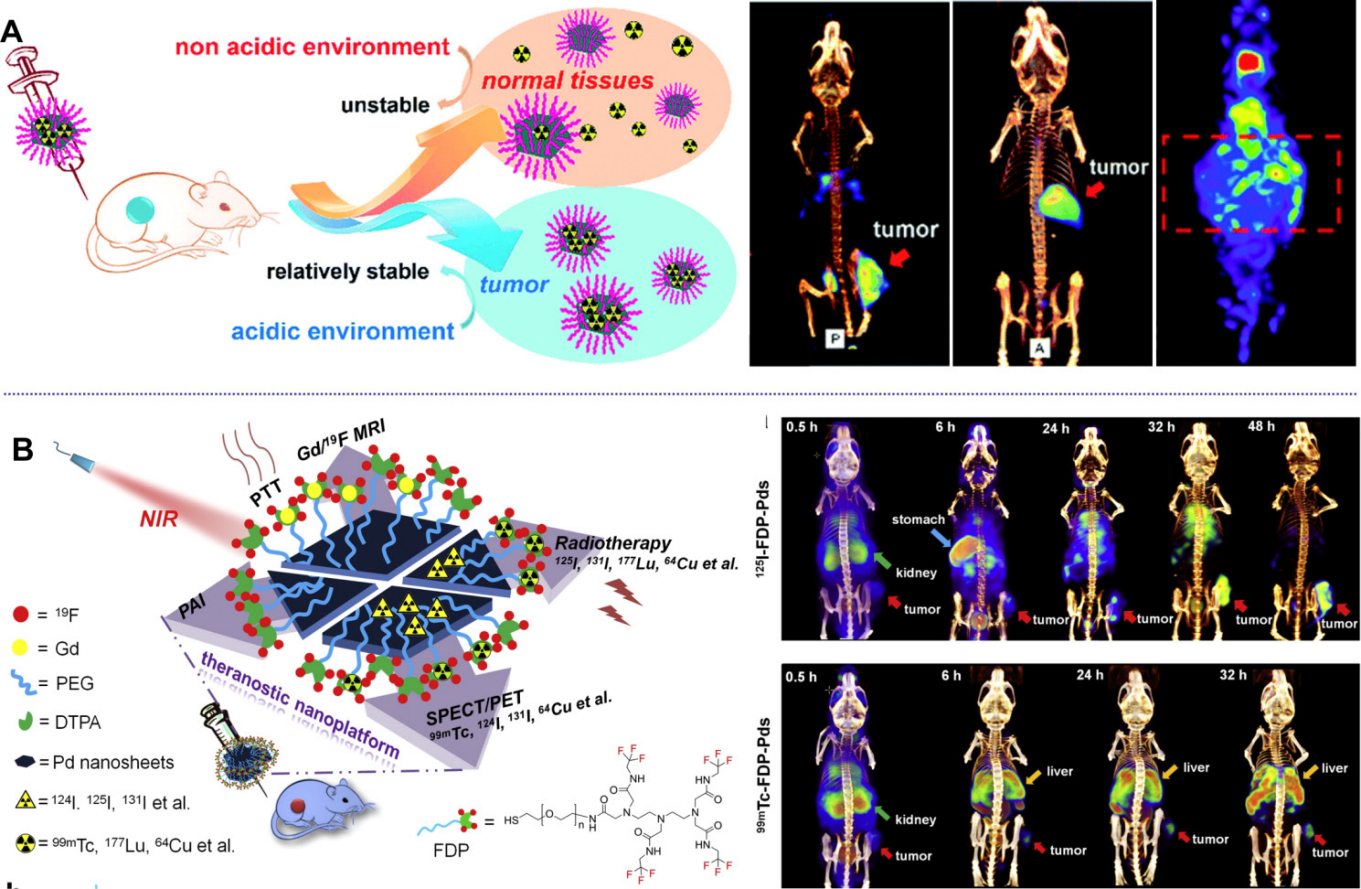
pH-sensitive radiolabeled Pd NSs for SPECT imaging in different tumor models, (**A**) radioactive iodide-labeled Pd NSs and (**B**) radioactive iodide and ^99m^Tc-labeled Pd NSs as a multifunctional theranostic platform. Adapted with permission from ref. 47. Copyright 2018, Royal Society of Chemistry; adapted with permission from ref. 48. Copyright 2018, Elsevier.

Different form the above-mentioned methods for labelling of radioisotopes, Kim et al. reported a direct labelling of ^125^I on the surface of Au NPs (^125^I-cRP-AuNP) through mixing ^125^I and Au NPs 46. ^125^I-cRP-AuNPs exhibit good stability in biological conditions and have been successfully applied in visualization of distribution of Au NPs by SPECT imaging. Inspired by the strong coordination effect between halide ions and Pd nanomaterials, which follows the order of I^-^ > Br^-^ > Cl^-^, several studies on the application of Pd-based nanomaterials in SPECT imaging have been reported by our group 47, 48. Radiolabeled Pd NSs were obtained by simple mixing of Pd NSs with ^125/131^I, resulting in a high labelling efficiency of 98% (Figure 3A) 47. Interestingly, the adsorption of radioiodine on the surface of Pd NSs shows pH-dependent behavior and better stability in acidic solution, which is an ideal theranostic platform for tumor imaging. After i.v. injection, high-quality SPECT images were obtained in subcutaneous and orthotopic tumor models. Moreover,^ 99m^Tc-labeled Pd NSs prepared by chelating with diethylentriamene pentaacetate (DTPA) exhibit excellent stability in physiological environments, and could provide complementary biological information together with ^125/131^I (Figure 3B) 48.

### CT imaging

Computed tomography (CT) is the most commonly used noninvasive clinical imaging modality, which presents internal anatomic structures of tissues based on the absorption of X-rays of different tissues. In order to enhance the contrast effect of soft tissues, contrast agents (e.g. iodinated molecules) have been administered before the imaging procedure. However, clinical iodinated agents often suffer rapid renal clearance and limited X-ray absorption capacity. To address these issues, application of nanomaterials with high X-ray attenuation coefficients as CT contrast agents have gained much attention for their favorable *in vivo* behaviors through optimization of composition, morphology, size and surface modification 49, 50. Among them, Au nanomaterials have become promising candidates as CT contrast agents because of their higher atomic number compared with iodine, prolonged blood circulation, enhanced permeability and retention (EPR) effect, and have greatly promoted the evolution of CT contrast agents 51, 52.

Pd nanomaterials have demonstrated good biocompatibility, long blood circulation half-life and high tumor accumulation. Although the atomic number of Pd is smaller than that of iodine, integration of Pd nanostructures with heavy elements (e.g. Au) has made them capable of serving as CT contrast agents. In 2014, our group first reported the application of Pd@Au nanoplates as CT contrast agents 15. Through the epitaxial growth of Au on the surface of 30-nm Pd NSs, Pd@Au nanoplates were synthesized with improved morphology and size, and even better tumor accumulation behavior. The absorption of X-rays exhibited linear positive correlation with the concentrations of Pd@Au nanoplates. After i.v. injection to tumor-bearing mice, enhanced CT imaging was obtained in the tumor site. Yang et al. also reported the application of Pd@Au nanoplates with partial coating of Au on Pd NSs as CT contrast agents 32. The CT contrast effect at the tumor site increased with time and reached a maximum at 24 h post injection with excellent delineation of the tumor. Except for Au coating, conjugation of iodinated CT contrast agents on the surface of Pd NSs may provide another promising way to develop novel CT contrast agents with high efficiency.

### MR imaging

Magnetic resonance (MR) imaging is another important noninvasive clinical modality for molecular and cellular imaging with high spatial resolution. MR imaging can provide detailed information about soft tissues and organs. To make precise diagnoses of specific diseases, MR imaging contrast agents have been commonly used to enhance the contrast effect of MR images by promoting relaxation of the surrounding water protons. Like other imaging technologies, combination with nanomaterials offers great promise for the further development of precise diagnosis in both clinical and preclinical research. Nanomaterials with paramagnetic or superparamagnetic properties can be applied in MR imaging through the optimization of size, morphology and surface modification 53, 54. For those nanostructures without magnetic properties, especially for non-magnetic noble metal nanostructures, such as Au NPs, this usually involves chelating with Gd-based compounds or integration of MR imaging contrast agents by formation of heterojunction structures, core-shell structures, and surface modification to realize the enhanced contrast of MR imaging 55.

**Figure 4:**
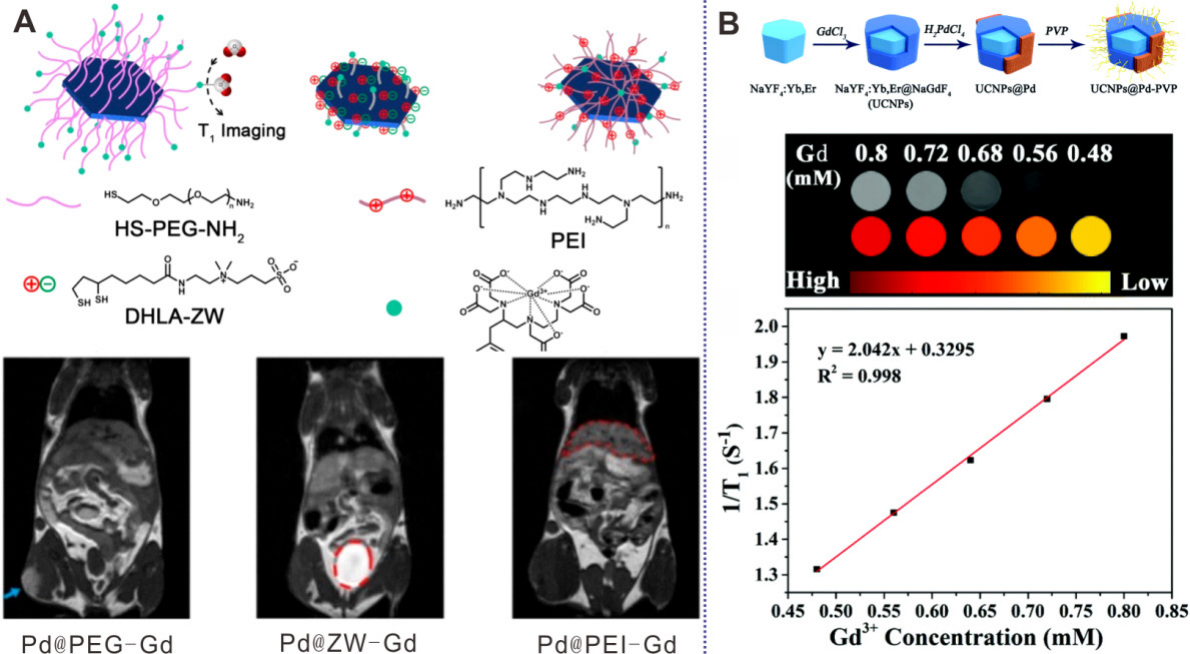
(**A**) Pd NSs with different surface modification for MRI imaging. (**B**) UCNPs@Pd-PVP nanocomposites for T1-weighted MRI imaging. Adapted with permission from ref. 56. Copyright 2018, Elsevier; adapted with permission from ref. 57. Copyright 2019, Royal Society of Chemistry.

Through coordination interaction, PEG, zwitterionic (ZW) ligands, and polyethylenimine (PEI) have been modified on the surfaces of Pd NSs with DTPA-Gd at the end of each molecule for real-time MR imaging to monitor the influence of surface coating on distribution of Pd NSs (Figure 4A) 56. As demonstrated by the results, Pd@PEG-Gd exhibited prolonged blood circulation time and high tumor accumulation, while Pd@ZW-Gd was rapidly excreted by renal clearance. Guo et al. also developed a multifunctional platform based on fluorinated DTPA-PEG-SH modified Pd NSs and successfully applied in Gd/F^19^ enhanced MR imaging guided cancer therapy 48. In addition, Zhao et al. reported the preparation of heterogeneous Pd NPs on the surfaces of upconversion nanoparticles (UCNPs) by a multi-step growth method (Figure 4B) 57. Owing to the presence of Gd^3+^, the composites were successfully applied as MR imaging contrast agents, providing a novel way to develop multifunctional nanoplatforms.

## Cancer therapy using Pd-based nanomaterials

Owing to the outstanding photothermal properties of Pd-based nanomaterials, their applications in cancer therapy primarily involve the PTT or combined therapies based on PTT. Photodynamic therapy, chemotherapy, radiotherapy and immunotherapy can all be combined with PTT to realize remarkable synergetic effects. As discussed above, Pd-based nanomaterials can also act as nanocarriers with high loading efficiency. Thus, various Pd-based theranostic nanoplatforms have been constructed by diverse surface modifications. In addition, Pd-based nanomaterials exhibit high catalytic activity and can contribute to the in-situ synthesis or activation of prodrugs for precise cancer chemotherapy.

### Photothermal therapy

PTT, is a newly developed and promising cancer treatment strategy, in which cancer cells are ablated by the heat generated from an optically absorbing agents upon laser irradiation, exhibiting minimal side effects, high specificity and controllability. Nanomaterial-mediated PTT has attracted much attention since the successful demonstration of Au nanoshells by Halas and coworkers in 2003 58. Thus, the development of ideal photothermal agents with strong NIR light absorption, high photothermal conversion efficiency and good biocompatibility has been critical for the potential clinical application of PTT. Consistent with PA imaging, the light absorption properties of nanomaterials become the starting point for PTT application 23. It is noteworthy that noble metal nanomaterials have attracted broad interests in the structure-spectrum relationship, owing to the development of synthetic techniques and facile surface modification strategies 59, 60. In the past two decades, Au nanomaterials have played a key role in promoting the advances of PTT 61. However, the anisotropic morphology of Au nanomaterials cannot be well maintained upon laser irradiation, resulting in decreased NIR absorption 62.

Compared with Au nanomaterials, Pd nanomaterials have shown obvious advantage in photothermal stability and photothermal conversion efficiency. In 2011, our group first reported the successful application of ultrathin hexagonal Pd NSs in PTT 10. The optical absorption peaks vary with the size of Pd NSs in the NIR region, making Pd NSs promising candidates for PTT. Since then, the biomedical applications of Pd-based nanomaterials have been attracting increasing interests. Pd NSs exhibit comparable extinction coefficients, but higher photothermal conversion efficiency and superior photothermal stability under NIR laser irradiation. Later, we successfully synthesized Pd corolla with increased apparent thickness by an etching growth strategy through the introduction of Fe^3+^ 13. Compared with Pd NSs with single-domain, Pd corolla exhibits much higher intracellular uptake efficiency, as well as *in vitro* PTT effect. We also demonstrated another effective method by coating silica on Pd NSs to achieve increased intracellular uptake 17. After surface amination, the uptake efficiency could be increased by 13 times. In addition, through surface regrowth second metal layer, such as Ag, Au, and Pt, the photothermal stability of Pd-based nanomaterials was greatly improved 14-16.

**Figure 5:**
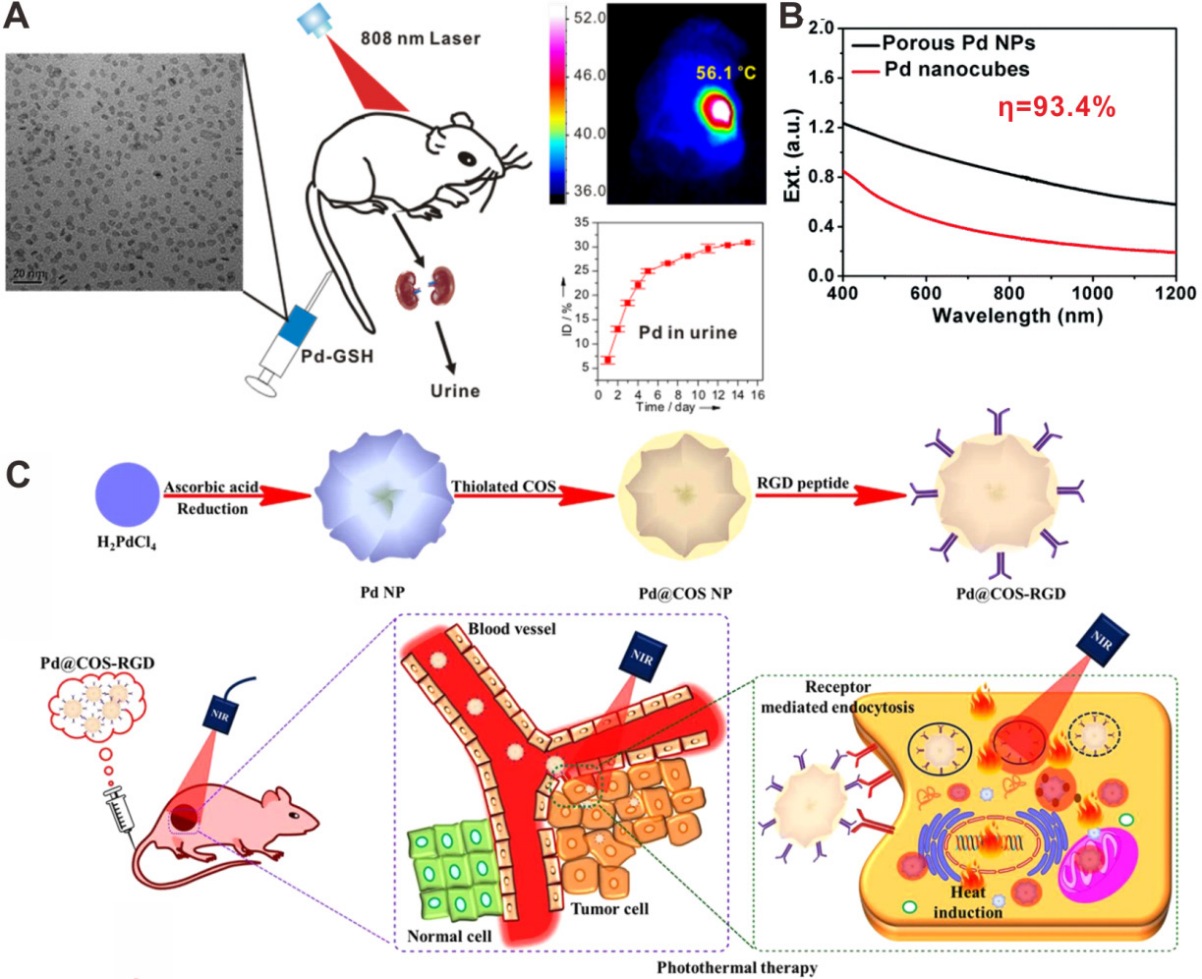
(**A**) Renal clearable ultra-small Pd NSs for PTT of cancer. (**B**) Optical spectra of porous Pd NPs and Pd nanocubes. (**C**) Chitosan oligosaccharide-coated Pd NPs for PPT of cancer. Adapted with permission from ref. 63. Copyright 2014, Wiley-VCH; adapted with permission from ref. 18. Copyright 2014, Royal Society of Chemistry; adapted with permission from ref. 20. Copyright 2018, Springer Nature.

In order to further expedite the application of Pd nanomaterials in cancer therapy, ultrasmall Pd NSs with an average particle size of 4.4 nm were successfully synthesized with strong NIR light absorption ability and high photothermal conversion efficiency (52.0%) (Figure 5A) 63. After surface functionalization with reduced glutathione (GSH), Pd-GSH exhibit prolonged blood circulation time and high accumulation in tumors, which can efficiently ablate tumors with NIR laser at a low power density of 1 W/cm^2^. Importantly, Pd-GSH could be cleared from the body through the renal excretion route after i.v. injection. Later, the influence of size and surface modification on the *in vivo* behavior of Pd NSs were systematically studied 11, 64. Pd NSs with thiol PEGylation and size smaller than 30 nm exhibited excellent performance *in vivo*. Utilizing 30 nm Pd NSs as seeds, Pd@Au nanoplates with similar size and shape were successfully synthesized 15. Compared with Pd NSs, the long-term stability of Pd@Au nanoplates were improved. With PEGylation, Pd@Au-PEG have shown relatively long blood circulation and the tumor accumulation through the EPR effect could reach as high as 79%ID/g 24 h after i.v. injection. More importantly, effective photothermal ablation of tumors could be achieved upon 808 nm laser irradiation at a power density of only 0.5 W/cm^2^. It is noteworthy that the adsorbed carbon monoxide (CO) on the surfaces of Pd NSs could also effectively enhance the EPR effect by NIR trigged release of CO for increasing vascular permeability 65. Recently, Wu and coworkers reported a cell-penetrating peptide (TAT) modification strategy to enhance the perinuclear accumulation of Pd NS. Upon mild laser irradiation, TAT modified Pd NS (Pd-TAT) could effectively enter the intranuclear from the perinuclear, resulting in ablation of primary tumor and inhibition of cancer metastasis 66.

Apart from 2D Pd-based nanomaterials, Pd nanomaterials with various morphologies and components have been reported as photothermal agents for PTT application. For instance, Yan and coworkers synthesized porous Pd NPs with strong NIR absorption (Figure 5B) 18. The photothermal conversion efficiency of porous Pd NPs reached as high as 93.4%, and cancer cells could be effectively ablated by a NIR laser. Liu et al. reported polypyrrole-coated flower-like Pd NPs (Pd NPs@PPy) as novel photothermal agents for PTT applications 19. The integration of polypyrrole endowed the composites with enhanced NIR light absorption, higher photothermal conversion efficiency and better compatibility. Moreover, porous Pd NPs showed high photostability upon laser irradiation 20. After modified with chitosan oligosaccharide (COS) and RGD peptide, Pd@COS-RGD NPs could be effectively uptaken by cells, and the accumulation in the tumor site was also increased, thus enhancing the PTT effect (Figure 5C). He and coworkers reported that incorporation of hydrogen into the lattice of Pd could effectively enhance the NIR absorption of Pd nanocubes, which were demonstrated as novel photothermal agents 33.

### Combined photothermal therapy and photodynamic therapy

**Figure 6:**
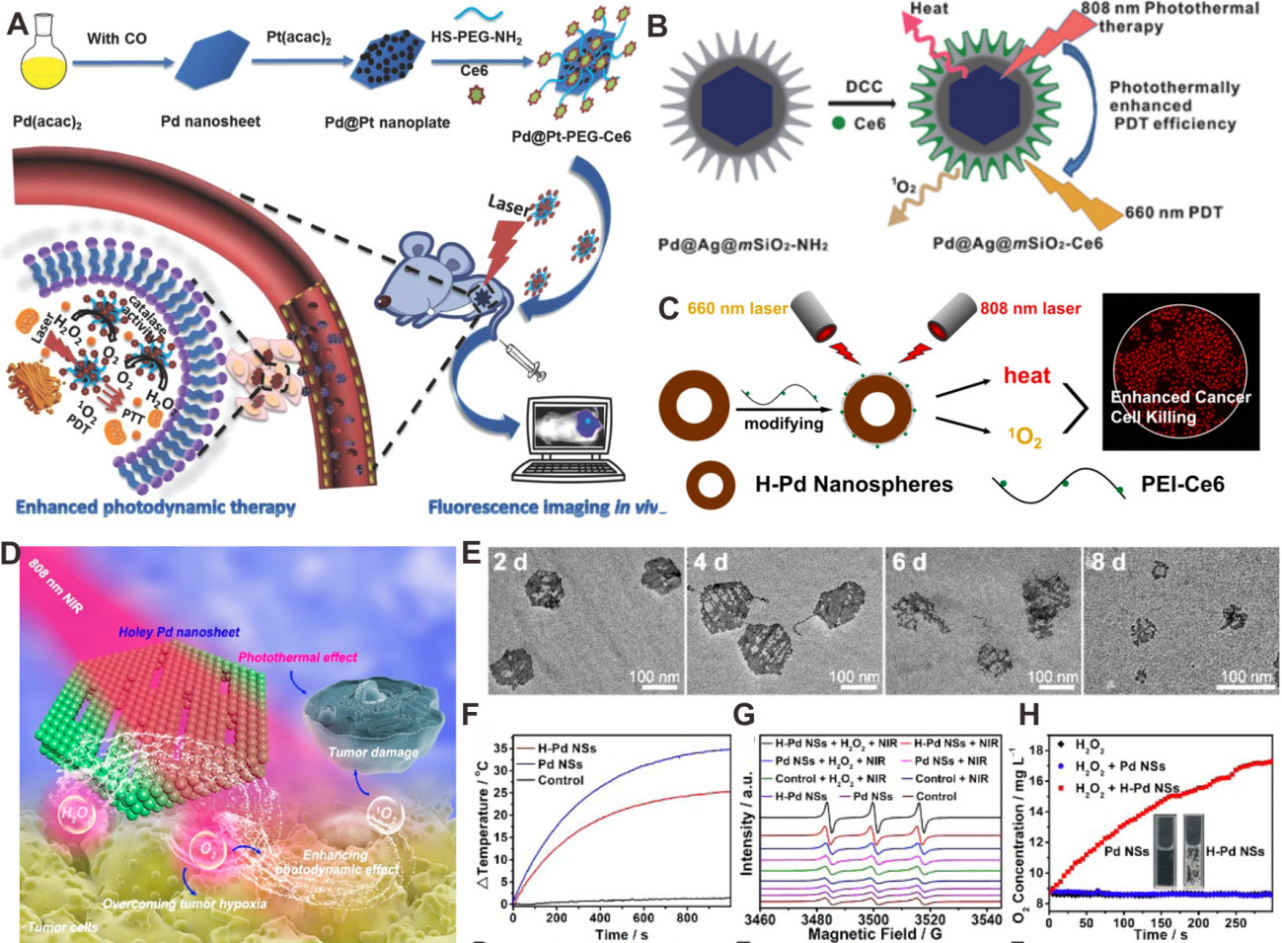
(**A**) Schematic representation of the synthesis and application of Pd@Pt-PEG-Ce6 for combined cancer therapy. (**B**) Construction of Pd@Ag@mSiO_2_-Ce6 for combined cancer therapy. (**C**) Hollow Pd nanospheres for combined cancer therapy. (**D**) Schematic illustration of combined cancer therapy using H-Pd NSs. (**E**) Degradation behavior of H-Pd NSs in simulated body fluid. (**F**) Photothermal effect of Pd NSs and H-Pd NSs. (**G**) ESR spectra and (**H**) O_2_ concentration of Pd NSs and H-Pd NSs under different conditions. Adapted with permission from ref. 16. Copyright 2018, Wiley-VCH; adapted with permission from ref. 70. Copyright 2013, American Chemical Society; adapted with permission from ref. 22. Copyright 2018, American Chemical Society; adapted with permission from ref. 72. Copyright 2020, American Chemical Society.

Similar to PTT, photodynamic therapy (PDT) is a cancer treatment based on the response of reagents to light irradiation. In PDT, the photosensitizers (PSs) deliver the power derived from light absorption to surrounding oxygen molecules (O_2_) to produce reactive oxygen species (ROS) to effectively destroy cancer cells 67. However, the inherent hypoxic nature of tumors is detrimental to PSs that rely on O_2_ to produce cytotoxic ROS. Meanwhile, depletion of O_2_ during PSs-mediated PDT also results in tumor hypoxia 68. So far, many strategies have been developed to overcome tumor hypoxia and improve PDT efficacy. For instance, nanomaterials with catalase (CAT)-like activity were used as catalysts for production of O_2_ *in situ* to relieve tumor hypoxia 69. Our group reported the development of a photosensitizer-Pd@Pt nanosystem (Pd@Pt-PEG-Ce6) based on Pd@Pt nanoplates (Figure 6A) 16, which severed as an efficient delivery platform for Ce6. Meanwhile, Pd@Pt nanoplates could trigger the decomposition of endogenous hydrogen peroxide (H_2_O_2_) to produce O_2_ and subsequently increase the O_2_ content in the tumor, further resulting in a significant improvement in PDT efficiency. In addition, the photothermal effect of Pd@Pt nanoplates also enhanced the PDT performed by Pd@Pt-PEG-Ce6 by increasing the catalytic activity.

Unlike PDT, PTT is a simple technique that is independent of O_2_ concentration, and the heat generated by PTT can increase the cell membrane permeability as well as the blood flow in the tumor region. Hence, combination of PTT and PDT can greatly improve the tumor therapy efficiency via a synergistic effect. Our group reported a combined PTT-PDT nanoplatform based on Pd@Ag@mSiO_2_-Ce6 NPs (Figure 6B) 70. The photothermal effect of Pd@Ag nanoplates enhanced the cellular internalization of Pd@Ag@mSiO_2_-Ce6 NPs and further improved the efficiency of PDT. In addition, Chen et al. developed a Pd-PEI-Ce6 nanocomplex for PTT-PDT synergistic therapy 71. Ce6 and Pd NSs served as the PDT and PTT agents, respectively, and PEI acted as the linker between Ce6 and Pd NSs. It was found that improved anticancer effect of combined PTT-PDT could be achieved. Besides, a new type of hollow Pd (H-Pd) nanospheres has been successfully synthesized (Figure 6C) 22. The H-Pd nanospheres exhibited excellent stability, and the absorption intensity and photothermal performance did not decrease even after 6 months when kept at room temperature. The combination of hollow Pd (H-Pd) nanospheres with Ce6 (Pd@Ce6) exhibited good photothermal conversion ability and high singlet oxygen (^1^O_2_) generation efficiently for PTT-PDT synergistic cancer therapy.

Recently, Liu and coworkers reported novel degradable holey Pd NSs (H-Pd NSs) obtained by stirring fresh Pd NSs in ethanol for 10 days to form 1D nanopores 72. H-Pd NSs maintained the photothermal capacity of Pd NSs (Figure 6D-6F). Interestingly, H-Pd NSs could produce ^1^O_2_ like PSs (Figure 6G). Moreover, H-Pd NSs also catalyzed the decomposition of H_2_O_2_ to generate O_2_, which further increased the yield of ^1^O_2_ (Figure 6H). Therefore, H-Pd NSs have become an ideal therapeutic platform for PTT-PDT synergistic therapy. Apart from that, by integration of Pd collora with indocyanine green (ICG) to form PdCs-HSA-ICG, both photothermal and photodynamic properties were successfully achieved for combined PTT-PDT cancer treatment 73.

### Combined photothermal therapy and chemotherapy

Chemotherapy is one of the main clinical methods of cancer treatment, but there are many limitations, such as poor water solubility of drugs, inferior pharmacokinetics, and inefficient accumulation in tumor site and drug resistance. Many researches have indicated that the combination of PTT and chemotherapy can solve these problems by a synergistic effect. PTT can enhance the cellular uptake of drugs by increasing the cell membrane permeability and can help reverse multidrug resistance mechanisms such as drug efflux. Furthermore, PTT has also shown to be an external trigger for tumor-specific and spatiotemporally controlled drug release, realizing on-demand chemotherapy 74, 75.

**Figure 7:**
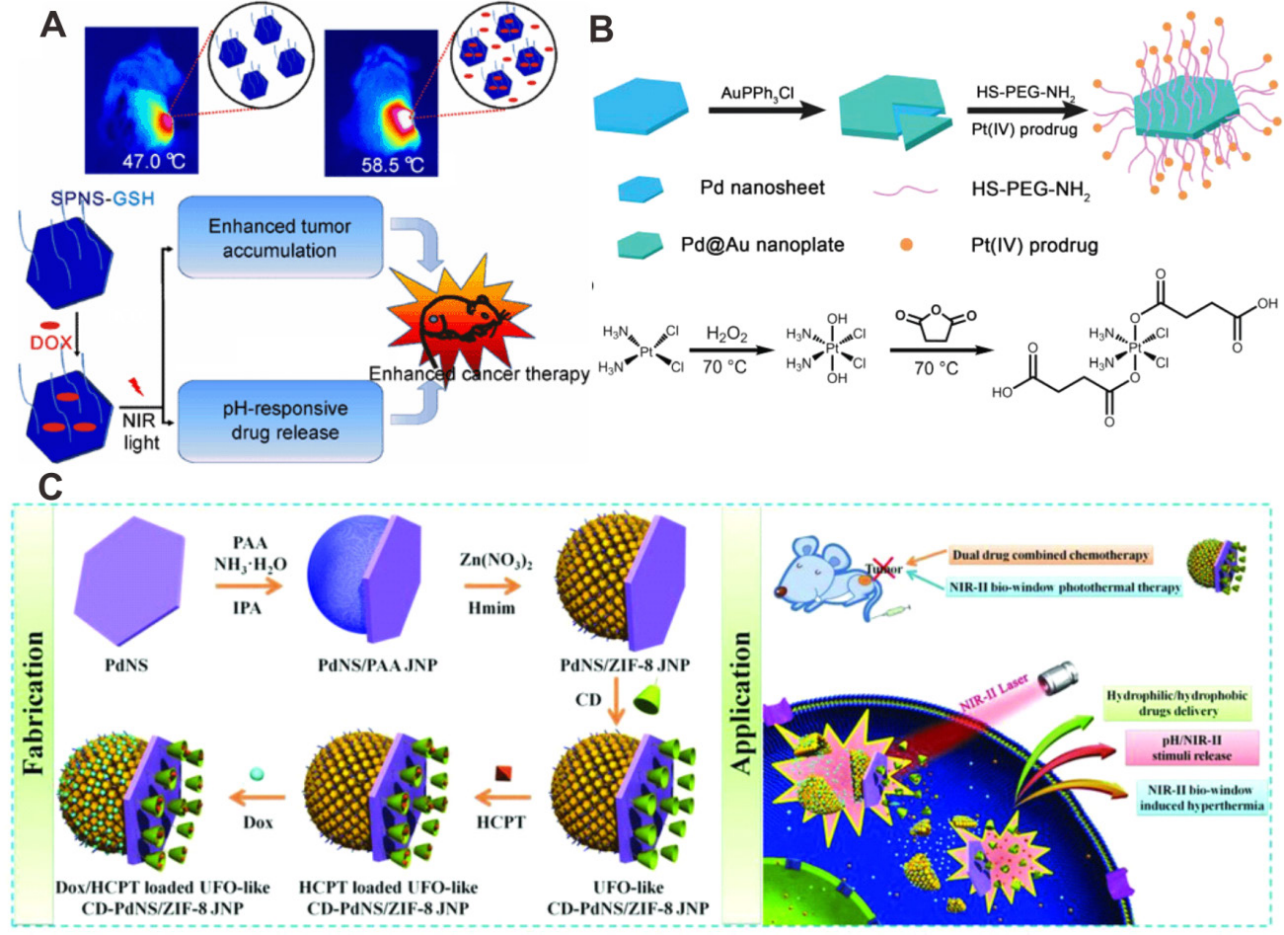
(**A**) Direct adsorption of DOX on the surfaces of Pd NSs for combined cancer therapy. (**B**) Synthesis of prodrug conjugated Pd@Au nanoplates for combined cancer therapy. (**C**) Synergistic anticancer application of DOX/HCPT co-loaded UFO-like Pd-based Janus NPs. Adapted with permission from ref. 76. Copyright 2015, Springer Nature; adapted with permission from ref. 77. Copyright 2016, Royal Society of Chemistry; adapted with permission from ref. 21. Copyright 2018, Wiley-VCH.

Recently, the application of Pd-based nanomaterials as drug delivery platforms has received much attention. There are two major ways for combining Pd-based nanomaterials with chemotherapeutic drugs: 1) direct drugs loading via coordinate bonds; 2) drug loading assisted by porous materials. Zheng et al. developed a multifunctional system for combining chemotherapy with PTT using ultrasmall Pd NSs by direct loading of doxorubicin (DOX) on the surfaces through Pd-N coordinate bonds (Figure 7A) 76. Later, Pd@Au nanoplates were combined with a platinum (IV) prodrug to form a versatile nanocomposite (Pd@Au-PEG-Pt) via standard amide coupling reactions 77. Pt(IV) prodrugs could readily be reduced by physiological reductants (e.g. ascorbic acid or glutathione) to produce hydrophilic and cytotoxic Pt (II) in cancer cells (Figure 7B). Because of the high tumor accumulation (29% ID/g), Pd@Au-PEG-Pt could achieve good therapeutic efficiency upon laser irradiation at relatively low power density.

As inorganic nanomaterials, mesoporous silicon exhibits unique advantages in the field of controlled drug release. In 2012, combination of Pd NSs with mesoporous silicon for drug loading was synthesized to achieve effective PPT-chemotherapy synergistic therapy 78. Composites based on Pd@Ag nanoplates and mesoporous silica were also reported for synergistic cancer therapy 79. In addition, metal-organic framework (MOF)-based nanoplatforms are also alternative transport vehicles due to their large drug loading capability. For example, ZIF-8 has become one of the most widely used MOFs in biomedicine. Pd@Au NPs and DOX encapsulated by ZIF-8 has been reported for synergistic PTT-chemotherapy 80. Later, Chen et al. developed a new theranostic nanoplatform (DOX/Pd@ZIF-8@PDA) by encapsulating 2D Pd NSs and DOX, taking advantage of the excellent photothermal effect of Pd NSs and drug loading capability of ZIF-8 81. These nanoplatforms both showed excellent photothermal effect and could perform smart drug release stimulated by laser irradiation and the tumor microenvironment. Recently, Wang et al. reported the preparation of an unidentified flying object (UFO)-cyclodextrin-Pd NSs/ZIF-8 Janus NPs (CD-PdNs/ZIF-8 JNP), which could deliver both hydrophobic 10-hydroxycamptothecin (HCPT) and hydrophilic DOX (Figure 7C) 21. The responsive release of the loading cargoes was achieved by simultaneous stimulation of pH and laser, followed by significant tumor inhibiting effect. Recently, Wu and coworkers developed a Pd NSs-based hydrogel (DOX@Pd Gel) by crosslinking Pd NSs with PEG as novel drug delivery platforms for synergistic PTT-chemotherapy. DOX@Pd Gel exhibited controllable release of DOX under NIR laser irradiation, and could effectively inhibit lung tumor metastasis 82.

Combined PTT-chemotherapy has also been applied for other diseases. For instance, rheumatoid arthritis is a stubborn disease caused by an autoimmune process, and the current clinical drug is methotrexate (MTX). However, the poor selectivity to inflammatory cells severely limits the use of MTX. To address this issue, a nanotherapeutic agent, Pd-Cys@MTX@RGD was synthesized, which could greatly reduce the toxicity of MTX and perform controlled release of MTX upon laser irradiation 83. After treatment, interleukin-1β (IL-1β) and vascular endothelial growth factor-induced inflammation could be effectively inhibited. In addition, the expression of proinflammatory cytokines could also be suppressed.

### Combined photothermal therapy and radiotherapy

Radiotherapy (RT), as one of the most frequent and effective cancer treatment modalities, involves the use of high-energy ionizing radiation (i.e., X-ray and γ-ray) to kill tumor cells by direct interaction with biomolecules, and indirect oxidative damage by free radicals generated from the radiolysis of water molecules. However, normal tissues surrounding the tumors often suffer serious damage under high dose ionizing radiation because of nonspecific interactions. Thus, radiosensitizers have been employed to enhance the effect of RT under low-dose ionizing radiation by increasing the radiosensitivity of tumor cells 84. Similar to CT contrast agents, nanomaterials composed of high atomic number metals have been developed as potential radiosensitizers to enhance the efficiency of RT. Noble metal nanomaterials, mainly Au and Pt, have been widely applied as radiosensitizers for cancer therapy 85, 86. Another obstacle limiting the therapeutic effect of RT is the hypoxic character of solid tumors, resulting in hypoxia-induced RT tolerance. Strategies for exogenous O_2_ import and endogenous production of O_2_ with the assistance of nanomaterials have been developed to obtain elevated O_2_ concentration 87. Moreover, remarkable synergistic effects were achieved by combing RT with other therapeutic modalities. Among them, PTT represents a promising candidate, because it can relieve tumor hypoxia by increasing the tumor blood flow and inhibit the repair of damaged DNA.

**Figure 8:**
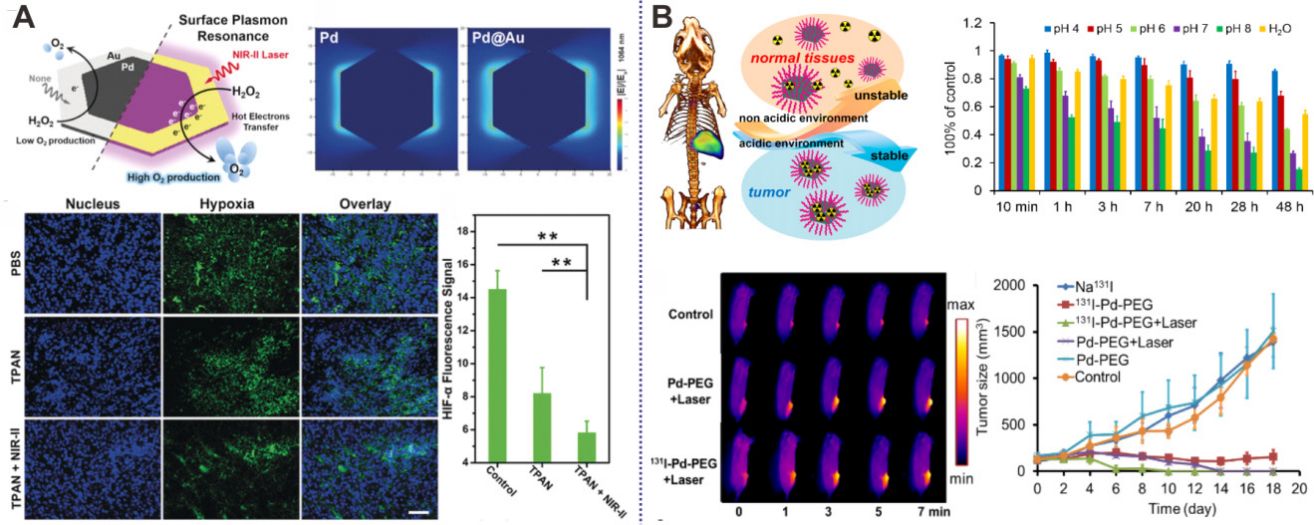
(**A**) Pd@Au nanoplates with continuous production of O_2_ by catalyzing the decomposition of endogenous H_2_O_2_ to overcome tumor hypoxia. (**B**) pH-responsive radiolabeled Pd NSs for combined cancer therapy. Adapted with permission from ref. 32. Copyright 2019, Wiley-VCH; adapted with permission from ref. 47. Copyright 2018, Royal Society of Chemistry.

Taking advantage of multi-functionalization of nanomaterials, Yang et al. confirmed that Pd@Au nanoplates with partial coating of Au on Pd NSs could overcome tumor hypoxia-induced RT tolerance (Figure 8A) 32. The exposed Pd could continuously promote the production of O_2_ by catalyzing the decomposition of endogenous H_2_O_2_. More importantly, the catalytic activity of Pd@Au nanoplates could be enhanced by the surface plasmon resonance effect induced by NIR-II laser irradiation. Utilizing the excellent absorption of NIR light, strong X-ray absorption and high tumor accumulation, Pd@Au nanoplates have been successfully applied in PTT-RT synergistic therapy guided by multimodal imaging. The integration of radioisotopes with nanomaterials has also been widely explored for combined cancer therapy 38. Since the radioisotope can directly produce ionizing radiation, efficient delivery and long-term retention of the radioisotope at the tumor site is of great importance. Our group reported the development of pH-sensitive multifunctional platform based on radiolabeled Pd NSs as mentioned above (Figure 8B) 47, 48. ^131^I could be easily labeled on the surface of Pd NSs for prolonged retention in tumor sites, effectively reducing the long-term toxicity caused by non-selective accumulation of radioisotopes. Together with the excellent photothermal effect of Pd NSs, remarkable synergistic therapeutic efficiency was achieved.

### Combined photothermal therapy and immunotherapy

Cancer immunotherapy has provided new approaches for cancer therapy by boosting the host immune system. Adaptive antitumor immune responses primed by immunotherapy can promote systemic immune surveillance and eliminate local and disseminated metastatic tumors. Different strategies for the activation and modulation of immune cells have been explored for cancer immunotherapy, including cancer vaccines, cytokine therapy, chimeric antigen receptor (CAR)-modified T cell therapy and immune checkpoint-blockade therapy 88-91. Recently, it was found that the combination of nanomaterials with immunomodulatory agents can modulate the tumor microenvironment, as well as trigger systemic antitumor immunity in overcoming tumor metastasis or recurrence 92.

**Figure 9:**
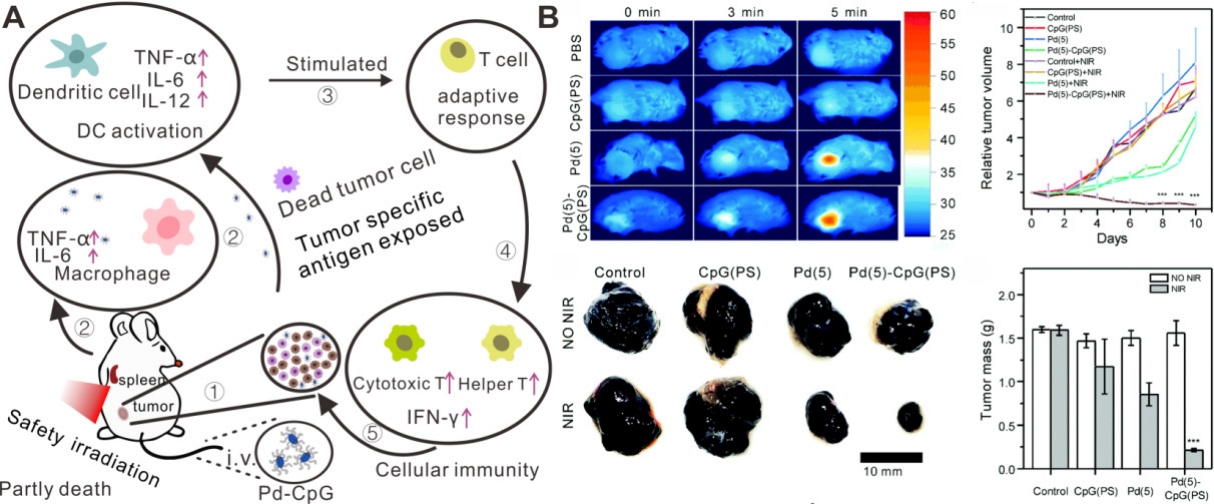
(**A**) Schematic illustration of the mechanism on the synergistic tumor inhibition of Pd-CpG. (**B**) *In vivo* combined cancer therapy of Pd-CpG. Adapted with permission from ref. 98. Copyright 2020, Royal Society of Chemistry.

While PTT can effectively eliminate the primary tumor, it usually fails to inhibit tumor metastasis and reoccurrence. The integration of PTT with immunetherapy involving the incorporation of photothermal agents with immunomodulatory agents has brought some inspiring results 74, 93. Enhanced anti-cancer effects have been successfully obtained by the combination of photothermal agents and immune checkpoint blockades 94 and immunoadjuvants 95-97. Recently, Pd NSs were reported as carriers of immunoadjuvant CpG oligodeoxynucleotides by our group 98. CpG recognized by Toll-like receptor 9 (TLR9) in the endosomes of antigen-presenting cells (APCs), exhibits potent immunostimulatory activities and has become a promising immunotherapeutic agent (Figure 9A). By modification with 3'-thiolated CpG on the surfaces of Pd NSs, CpG could be efficiently delivered to tumor sites. PTT based on Pd NSs could induce tumor cell death and release tumor-associated antigens, which could be effectively uptaken and presented by antigen-presenting cells with the assistance of CpG, resulting in increased tumor necrosis factor-α (TNF-α) and interleukin-6 (IL-6) production and enhanced cytotoxic T lymphocyte (CTL) activity, thus achieving enhanced cancer therapy (Figure 9B).

### Combined photothermal therapy and hydrogen therapy

Since the research of Dole et al. in 1975 with tumor-bearing mice, the therapeutic application of hydrogen (H_2_) has been identified as a promising cancer therapy strategy in clinical practice 99, 100. H_2_ can selectively scavenge reactive oxygen species, especially hydroxyl radicals (•OH) and peroxynitrite anions (ONOO^-^) without influence on normal metabolic oxidation/reduction reactions or cell signal transduction. In addition to tumor therapy, potential applications in anti-oxidant, anti-inflammatory, and anti-apoptotic scenarios have been studied 101-103. Compared with chemotherapeutic drugs, H_2_ can easily penetrate biological membranes and diffuse in tissues and cells. Current administration routes for H_2_ are mainly divided into gaseous form for inhalation and dissolved gas in liquids for oral intake. For cancer treatment, it is necessary to have a relatively high concentration of H_2_ at tumor sites. However, it is difficult to achieve targeted delivery and controlled release of H_2_ in specific areas because of the free diffusion of H_2_. In addition, the storage efficacy of H_2_ in water or saline is usually limited due to the low solubility. Hence, the development of new strategies for effective storage, targeted delivery and controlled release of H_2_ is of great importance for optimal hydrogen therapeutic efficacy.

**Figure 10:**
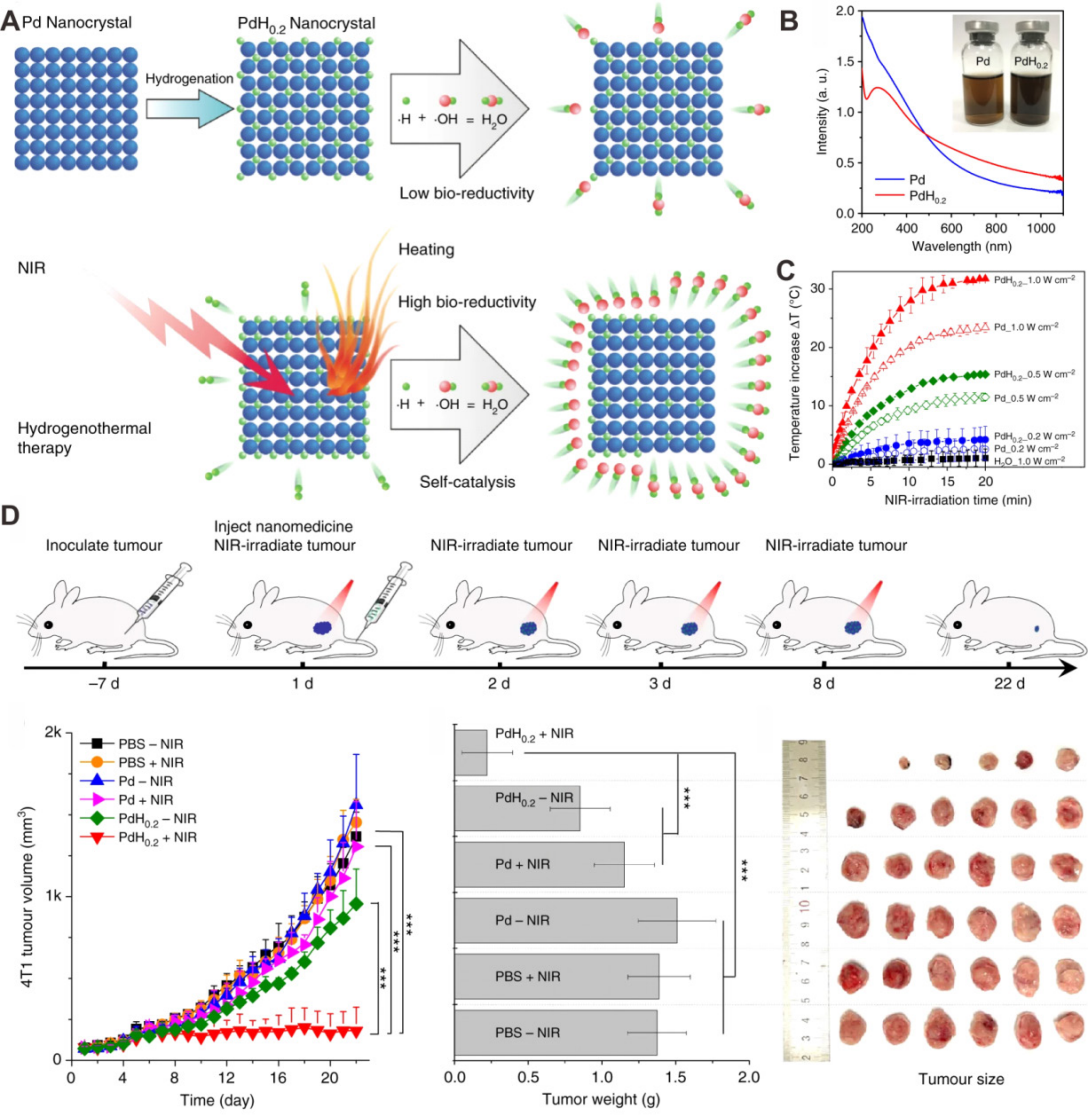
(**A**) Schematic illustration showing the formation and laser-stimulated release of PdH_0.2_ nanocrystals for combined cancer therapy. (**B**) Optical spectra of Pd and PdH_0.2_ nanocrystals. (**C**) Photothermal effect of Pd and PdH_0.2_ nanocrystals. (**D**) *In vivo* combined cancer therapy of PdH_0.2_ nanocrystals. Adapted with permission from ref. 33. Copyright 2018, Springer Nature.

As described above, the development of nanotechnology has greatly promoted the diversification of nanomedicine for cancer therapy. Integration of H_2_ therapy and nanomaterials makes it be possible to achieve targeted delivery of H_2_, as well as controlled release of H_2_ under stimulation by the tumor microenvironment or external laser irradiation. Pd nanomaterials, which can incorporate of H_2_ into the Pd lattice, have attracted broad interests for their potential applications in H_2_ sensors, H_2_ storage and purification 104, 105. Pd hydride nanomaterials with different morphologies have demonstrated relatively high stability and electrocatalytic activity. More importantly, the optical absorption of PdHx in the NIR region is enhanced by the formation of Pd hydride, enabling their applications in biomedical areas. He and coworkers were the first to report the application of Pd hydride (PdH_0.2_) in synergetic hydrogenothermal cancer therapy. PdH_0.2_ nanocubes were synthesized by direct exposure of Pd nanocubes to H_2_, and kept the morphology and size of the parent Pd nanocubes 33. Compared with Pd nanocubes, PdH_0.2_ exhibited obvious enhanced absorption of NIR light and superior photothermal properties upon 808 nm laser irradiation (Figure 10A-C). The release of bioreductive H_2_ from PdH_0.2_ follows the typical profile of a photochemical reaction upon NIR laser irradiation. Interestingly, the released H_2_ has the capacity to modulate the abnormal level of ROS in normal cells. *In vivo* studies revealed that PdH_0.2_ nanocrystals had high tumoral targeting capability, providing a safe and effective way for targeted delivery of H_2_ to tumor sites (Figure 10D). Similarly, He et al. developed another Pd hydride theranostic platform based on a Pd metal-organic framework (PdH-MOF) by the coordination of Pd and porphyrin 106. PdH-MOF exhibited high H_2_ loading capacity and improved photothermal conversion efficiency. With sustained reductive H_2_ release performance and high tumor targeting behavior, PdH-MOF NPs were successfully applied for PA imaging guided hydrogenothermal cancer therapy.

### Prodrug activation for cancer chemotherapy

Pd-based catalysts are widely used in industry but less so in biological systems, mainly because the complexity of biological systems cannot meet the conditions of catalytic reaction 107-109. Although the utilization of Pd nanostructures as biological catalysts is challenging, numerous efforts have been devoted to apply Pd-based catalysts in many biological fields, including *in vivo* imaging, cell labeling and prodrug activation. In terms of *in vivo* imaging, Anh and coworkers reported that Pd (0), Pd (II) or Pd (IV) catalyzed non-fluorescence substances into fluorescent materials through depropargylation reaction for the detection of palladium species in zebrafish models 110. Davis et al. introduced a Pd-mediated Suzuki-Miyaura cross-coupling approach for living cell surface labelling 111.

**Figure 11:**
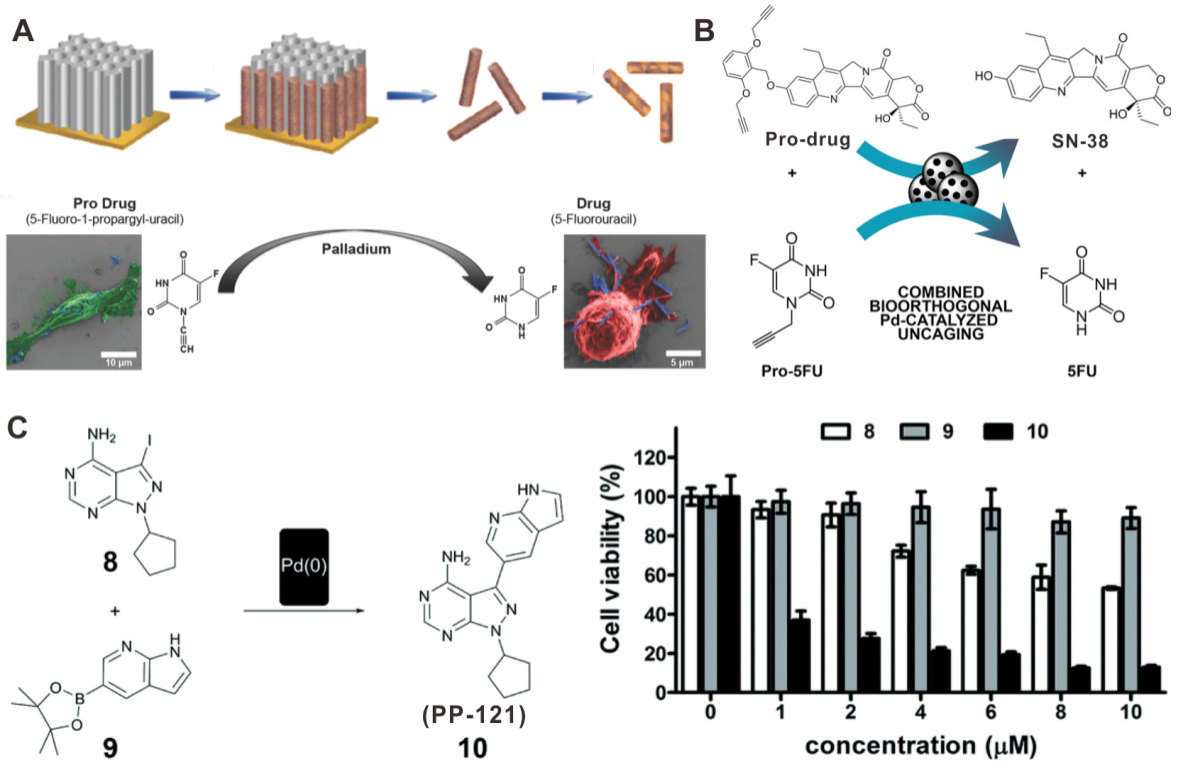
Illustration of Pd nanomaterials for prodrug activation and synthesis of toxic drugs *in vivo* for cancer chemotherapy. (**A**) The synthetic process of FePd NWs and activation of pro-5FU. (**B**) Schematic illustration of the activation of prodrug and pro-5FU by Pd devices. (**C**) Synthesis of PP-121 by Pd(0) catalysts and cytotoxicity tests. Adapted with permission from ref. 115. Copyright 2018, Wiley-VCH; adapted with permission from ref. 116. Copyright 2018, Wiley-VCH; adapted with permission from ref. 117. Copyright 2016, Royal Society of Chemistry.

Pd-based catalysts can also play prominent roles in the field of bioorthogonal catalysis by catalyzing the change of inert prodrugs into toxic drugs or by inducing the release of “caged” biomolecules into biological systems 112-114. For instance, hybrid FePt nanowires (FePd NWs) has been proposed for the catalytic activation of prodrug 5-fluoro-1-propionyl-uracil (Pro-5-FU) to 5-fluorouracil (5-FU) (Figure 11A) 115. The intraperitoneal injection of Pro-5-FU together with FePd NWs could significantly inhibit tumor growth without obvious side effects. Another example of prodrug activation by Pd nanomaterials is the release of active metabolite of irinotecan (SN-38). “Caged” SN-38 blocked by a novel alkyl group was prepared to notably reduce the cytotoxic activity of SN-38. The Pd devices could activate the release of SN-38 at desired locations and give rise to the production of 5-FU after intratumoral implanting (Figure 11B) 116. In addition, encapsulation of ultrathin Pd NSs in cancer cell derived vesicles (Pd-Exo) as new artificial biological devices was developed to achieve the selective delivery of Pd catalysts 113. Pd-Exo could be uptaken with high efficiency to achieve the specific release of the anticancer drug panobinostat, thereby leading to a new strategy for realizing precisely targeted chemotherapy.

*In situ* synthesis of bioactive reagents stimulated by *in vivo* bioorthogonal chemistry has also proved to be a promising strategy for improving cancer therapy. As a novel prodrug activation strategy, Pd NPs were encapsulated in a modular polymeric carriers to assist the synthesis of anticancer drug PP-121 from two nontoxic precursors, thus becoming the first case of *in situ* Pd-induced drug synthesis (Figure 11C) 117. Moreover, Pd NPs mediated fluorescent microspheres could also catalyze the synthesis of PP-121, while activating the precursor drugs of 5-FU in glioblastoma cells. The synthetic strategy of this dual anticancer drug exhibited better cancer therapeutic effect than the individual drugs.

## Biosafety profiles of Pd-based nanomaterials

**Figure 12:**
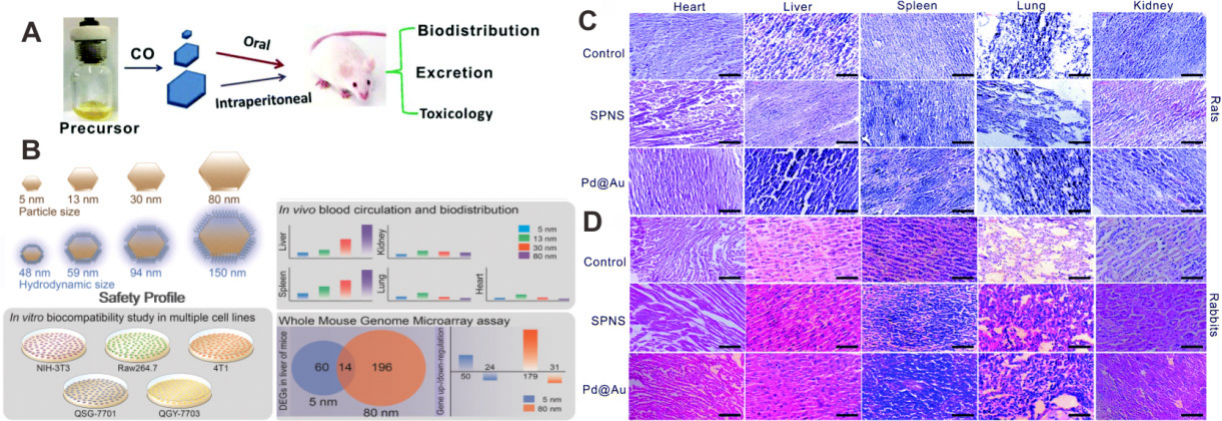
(**A**) Effect of size and administration routes on the biobehaviors of Pd NSs. (**B**) Safety profile of different-sized Pd NSs *in vitro* and *in vivo*. H&E images of (**C**) rats and (**D**) rabbits after i.v. injection of Pd NSs and Pd@Au nanoplates. Adapted with permission from ref. 118. Copyright 2017, Royal Society of Chemistry; adapted with permission from ref. 11. Copyright 2017, Springer Nature; adapted with permission from ref. 120. Copyright 2019, Royal Society of Chemistry.

Biological safety profiles have become a vital index for evaluating nanomaterials for their future clinical translational applications. The following aspects should be emphasized in these studies: 1) pharmacokinetics and bio-distribution; 2) metabolic and clearance mechanisms; 3) acute and long-term toxicity; 4) mechanisms behind pharmacological activity. For Pd-based nanomaterials, systemic studies on 2D Pd NSs have been carried out to demonstrate the safety profiles of Pd-based nanomaterials. The toxicity of Pd NSs after oral feeding or intraperitoneal injection was investigated in a mouse model, and no detectable lesions were observed in major organs confirmed by hematoxylin and eosin (H&E) staining study, indicating that different sizes of Pd NSs do not give rise to conspicuous side effects on normal tissues following diverse administration approaches (Figure 12A) 118. Another *in vivo* study suggested that 5 nm Pd NSs could easily escape from the reticuloendothelial system with prolonged blood half-life compared to larger Pd NSs, and could be cleared through renal excretion. 30 nm Pd NSs exhibited the highest tumor accumulation efficiency, whereas there was still also considerable retention in the liver and spleen (Figure 12B) 11. Moreover, Chen and coworkers found that GSH played a significant role in the renal clearance of Pd NSs 119. Surface modification with GSH or post injection of GSH could greatly promote the clearance of Pd NSs. Recently, Pd NSs with 1D nanopores were fabricated utilizing a surface etching strategy 72. Under physiological conditions, these Pd NSs exhibited degradable behavior, which contradicts the traditional concept of stability for noble metal materials and is of great significance to broaden the theranostic applications of Pd-based nanomaterials.

In addition to the mouse model, Li et al. have analyzed the change of biochemical indices and hematology markers caused by 5 nm Pd NSs and 30 nm Pd@Au nanoplates in rats and rabbits, respectively 120. All the results suggested that Pd NSs and Pd@Au nanoplates did not cause obvious side effects on blood chemistry and liver/renal function at the given dose. In addition, histological analysis of various organ tissues can be used to determine whether nanomaterials could cause tissue damage, inflammation or lesions, and is another way to clarify the biological safety of nanomaterials. In the work of Li et al., five representative organs (liver, spleen, lung, kidney and heart) were fixed, stained and analyzed 48 hours post-injection, and there were no significant histopathological abnormalities or lung lesions (Figure 12C and Figure 12D).

## Challenges and opportunities

In conclusion, the controlled synthesis and various modification strategies of nanomaterials can provide many opportunities for the rational design of multifunctional nanoplatforms for cancer theranostics. Among these novel nanomaterials, the progress of Pd-based nanomaterials has greatly advanced the applications of noble metal nanomaterials in biomedical fields. Compared with other noble metal nanostructures, Pd-based nanomaterials have unique advantages, such as good photothermal stability and biocompatibility, as well as high photothermal conversion efficiency, making them outstanding and promising in biomedicine. As mentioned above, after a decade of development, Pd-based nanomaterials, e.g., Pd NSs, Pd NPs, Pd@Au and Pd@Pt nanostructures have been extensively studied in multimodal imaging-guided cancer therapy. However, the challenges and prospective opportunities of Pd-based nanomaterials in the biomedical field still need to be discussed seriously, particularly as follows:

First, we have summarized the combinations of Pd-based nanomaterials with chemotherapy drugs or prodrugs as synergetic tumor therapeutic platforms, and several studies demonstrated that Pd-based catalysts could be applied as novel prodrug activating factors. However, few Pd nanomaterials are designed to make full use of the tumor microenvironment for sequential activation or enhancement of cancer treatment. And Pd-based nanomaterials, which can intensify the hypoxic microenvironment, are encouraging for boosting anti-cancer effects. Furthermore, the synergistic therapies still need to be further explored for continuous treatment of the remaining tiny lesions.

Next, Pd-based nanomaterials are rarely investigated as biosensors for the detection of tumor biomarkers. Tumor biomarkers play important roles in tumor theranostics because they can be exploited for early cancer diagnosis, as well as evaluation of tumor progression. Thus, detection of tumor biomarkers with high sensitivity is essential for efficient cancer therapy and evaluation of therapeutic effect. Pd NWs 121 and Pd@Au nanoplates 122 exhibited high fluorescence quenching ability, which could significantly quench the fluorescence signal of fluorescent dye labeled single-stranded DNA probes. The fluorescence signal could be recovered in the presence of target DNA, leading to highly sensitive detection of target DNA. However, highly efficient detection platforms and their working mechanisms need to be further investigated. It is believed that Pd-based biosensing platforms can contribute to discriminating the subtypes of tumors and designing individual therapeutic plans.

The application of Pd-based nanomaterials in theranostics should be further expanded. Treatment of resistant bacteria has become a hot topic in recent years. Noble metal-based nanomaterials with exposure of a specific crystal facet have shown promise as alternative enzyme mimics. Some studies have confirmed that Pd nanocrystals have oxidase or peroxidase-like activity for antibacterial treatment by elevating the production of ROS 123, 124. A recent study demonstrated that enzyme-catalytic Pd@Pt nanoplates could act as sonodynamic therapy sensitizers against drug-resistant bacteria to eradicate methicillin-resistant staphylococcus aureus-induced myositis 125. Because of the correlation between the mechanisms of antibacterial and anticancer processes, the existing cancer theranostic nanoplatforms discussed above may provide crucial clues for the design and manipulation of Pd-based antimicrobial agents.

Last but not the least, the biological safety of Pd-based nanomaterials is still a key point for advancing their practical applications. Some studies have shown that Pd NSs with different sizes exhibited good biocompatibility in mice, rats and rabbits. In particular, 5 nm Pd NSs exhibit long blood half-life and can be easily cleared out through renal excretion, while the larger Pd NSs mainly accumulate in the liver and spleen without obvious toxicity. Besides controlling the size to manipulate their performance, surface modification (e.g. cell membrane coating and active tumor-targeting ligand conjugation) is an alternative approach to optimize the*in vivo* behavior of Pd NSs. Although some progresses in biocompatibility studies of Pd nanomaterials have been achieved, there is still a long way to go for clinical translation. The long-term biosafety, pathways of metabolism, toxicity on embryos, etc. are all the essential questions waiting for detailed answers.

## Figures and Tables

**Table 1:** Summary of Pd-based nanomaterials for cancer imaging

Type of imaging	Materials	Reference
Photoacoustic imaging	Pd NSs	11, 31
	Pd@Au nanoplates	15, 32
	PdH_0.2_ cubes	33
	Pd@COS-RGD	20
	DOX/Pd@ZIF-8@PDA	81
	PdH-MOF	106
SPECT imaging	[^131^I]PHPdNPs-DOX	45
	^125^I-Pd-PEG	47
	^99m^Tc-FDP-Pds, ^125^I-FDP-Pds	48
CT imaging	Pd@Au-PEG	15, 32
MR imaging	Pd@PEG-Gd, Pd@PEI-Gd, Pd@ZW-Gd	56
	UCNPs@Pd-PVP	57
	Gd-FDP-Pds	48

**Table 2:** Summary of Pd-based nanomaterials for cancer therapy

Type of therapy	Materials	Reference
PTT	Pd NSs	10, 11, 63
	Pd collora	13
	Pd@Ag nanoplates	14
	Pd@Au nanoplates	15
	Pd@SiO_2_	17
	Porous Pd NPs	18
	Pd NPs@PPy	19
	Pd@COS-RGD	20
	Pd NS-CO	65
	Pd-TAT	66
PTT & PDT	Pd@Pt-PEG-Ce6	16
	H-Pd@Ce6	22
	Pd@Ag@mSiO_2_-Ce6	70
	Pd-PEI-Ce6	71
	H-Pd NSs	72
	PdCs-HSA-ICG	73
PTT & Chemotherapy	PdNS/ZIF-8 JNPs	21
	Dox-loaded 8dc-Pd NPs	75
	SPNS-DOX	76
	Pd@Au-PEG-Pt	77
	HMSS-NH_2_ /DOX@Pd	78
	Pd@Ag@sSiO_2_@mSiO_2_-DihBen/DOX	79
	DOX/Pd@Au@ZIF-8	80
	DOX/Pd@ZIF-8@PDA	81
	DOX@Pd Gel	82
	Pd-Cys@MTX@RGD	83
PTT & RT	[^131^I]PHPdNPs-DOX	45
	^131^I-Pd-PEG	47, 48
PTT & immunotherapy	Pd-CpG	94
PTT & hydrogen therapy	PdH_0.2_ nanocubes	33
	PdH-MOF	106

## References

[1] Siegel RL, Miller KD, Jemal A (2020). Cancer statistics, 2020. CA Cancer J Clin.

[2] Lammers T, Aime S, Hennink WE, Storm G, Kiessling F (2011). Theranostic nanomedicine. Acc Chem Res.

[3] Shi J, Kantoff PW, Wooster R, Farokhzad OC (2017). Cancer nanomedicine: progress, challenges and opportunities. Nat Rev Cancer.

[4] Huo S, Ma H, Huang K, Liu J, Wei T, Jin S (2013). Superior penetration and retention behavior of 50 nm gold nanoparticles in tumors. Cancer Res.

[5] Kim D, Shin K, Kwon SG, Hyeon T (2018). Synthesis and biomedical applications of multifunctional nanoparticles. Adv Mater.

[6] Chen A, Ostrom C (2015). Palladium-based nanomaterials: synthesis and electrochemical applications. Chem Rev.

[7] Chen M, Wu B, Yang J, Zheng N (2012). Small adsorbate-assisted shape control of Pd and Pt nanocrystals. Adv Mater.

[8] Chen X, Shi S, Wei J, Chen M, Zheng N (2017). Two-dimensional Pd-based nanomaterials for bioapplications. Sci Bull (Beijing).

[9] Jain PK, Huang X, El-Sayed IH, El-Sayed MA (2008). Noble metals on the nanoscale: optical and photothermal properties and some applications in imaging, sensing, biology, and medicine. Acc Chem Res.

[10] Huang X, Tang S, Mu X, Dai Y, Chen G, Zhou Z (2011). Freestanding palladium nanosheets with plasmonic and catalytic properties. Nat Nanotechnol.

[11] Chen M, Chen S, He C, Mo S, Wang X, Liu G (2017). Safety profile of two-dimensional Pd nanosheets for photothermal therapy and photoacoustic imaging. Nano Res.

[12] Dumas A, Couvreur P (2015). Palladium: a future key player in the nanomedical field?. Chem Sci.

[13] Huang X, Tang S, Yang J, Tan Y, Zheng N (2011). Etching growth under surface confinement: an effective strategy to prepare mesocrystalline Pd nanocorolla. J Am Chem Soc.

[14] Huang X, Tang S, Liu B, Ren B, Zheng N (2011). Enhancing the photothermal stability of plasmonic metal nanoplates by a core-shell architecture. Adv Mater.

[15] Chen M, Tang S, Guo Z, Wang X, Mo S, Huang X (2014). Core-shell Pd@Au nanoplates as theranostic agents for in-vivo photoacoustic imaging, CT imaging, and photothermal therapy. Adv Mater.

[16] Wei J, Li J, Sun D, Li Q, Ma J, Chen X (2018). A novel theranostic nanoplatform based on Pd@Pt-PEG-Ce6 for enhanced photodynamic therapy by modulating tumor hypoxia microenvironment. Adv Funct Mater.

[17] Tang S, Huang X, Zheng N (2011). Silica coating improves the efficacy of Pd nanosheets for photothermal therapy of cancer cells using near infrared laser. Chem Commun.

[18] Xiao J-W, Fan S-X, Wang F, Sun L-D, Zheng X-Y, Yan C-H (2014). Porous Pd nanoparticles with high photothermal conversion efficiency for efficient ablation of cancer cells. Nanoscale.

[19] Liu Y, Wang D-D, Zhao L, Lin M, Sun H-Z, Sun H-C (2016). Polypyrrole-coated flower-like Pd nanoparticles (Pd NPs@PPy) with enhanced stability and heat conversion efficiency for cancer photothermal therapy. RSC Adv.

[20] Bharathiraja S, Bui NQ, Manivasagan P, Moorthy MS, Mondal S, Seo H (2018). Multimodal tumor-homing chitosan oligosaccharide-coated biocompatible palladium nanoparticles for photo-based imaging and therapy. Sci Rep.

[21] Zhang L, Li S, Chen X, Wang T, Li L, Su Z (2018). Tailored surfaces on 2D material: UFO-like cyclodextrin-Pd nanosheet/metal organic framework Janus nanoparticles for synergistic cancer therapy. Adv Funct Mater.

[22] Liu Y, Ding L, Wang D, Lin M, Sun H, Zhang H (2018). Hollow Pd nanospheres conjugated with Ce6 to simultaneously realize photodynamic and photothermal therapy. ACS Appl Bio Mater.

[23] Fu Q, Zhu R, Song J, Yang H, Chen X (2019). Photoacoustic imaging: contrast agents and their biomedical applications. Adv Mater.

[24] Weber J, Beard PC, Bohndiek SE (2016). Contrast agents for molecular photoacoustic imaging. Nat Methods.

[25] Liu Y, Bhattarai P, Dai Z, Chen X (2019). Photothermal therapy and photoacoustic imaging via nanotheranostics in fighting cancer. Chem Soc Rev.

[26] Nie L, Chen X (2014). Structural and functional photoacoustic molecular tomography aided by emerging contrast agents. Chem Soc Rev.

[27] Sun C, Wen L, Zeng J, Wang Y, Sun Q, Deng L (2016). One-pot solventless preparation of PEGylated black phosphorus nanoparticles for photoacoustic imaging and photothermal therapy of cancer. Biomaterials.

[28] Li W, Chen X (2015). Gold nanoparticles for photoacoustic imaging. Nanomedicine.

[29] De La Zerda A, Zavaleta C, Keren S, Vaithilingam S, Bodapati S, Liu Z (2008). Carbon nanotubes as photoacoustic molecular imaging agents in living mice. Nat Nanotechnol.

[30] Yang K, Zhu L, Nie L, Sun X, Cheng L, Wu C (2014). Visualization of protease activity *in vivo* using an activatable photo-acoustic imaging probe based on CuS nanoparticles. Theranostics.

[31] Nie L, Chen M, Sun X, Rong P, Zheng N, Chen X (2014). Palladium nanosheets as highly stable and effective contrast agents for *in vivo* photoacoustic molecular imaging. Nanoscale.

[32] Yang Y, Chen M, Wang B, Wang P, Liu Y, Zhao Y (2019). NIR-II driven plasmon-enhanced catalysis for a timely supply of oxygen to overcome hypoxia-induced radiotherapy tolerance. Angew Chem Int Ed.

[33] Zhao P, Jin Z, Chen Q, Yang T, Chen D, Meng J (2018). Local generation of hydrogen for enhanced photothermal therapy. Nat Commun.

[34] Zhao L, Zhu J, Cheng Y, Xiong Z, Tang Y, Guo L (2015). Chlorotoxin-conjugated multifunctional dendrimers labeled with radionuclide ^131^I for single photon emission computed tomography imaging and radiotherapy of gliomas. ACS Appl Mater Interfaces.

[35] Polyak A, Ross TL (2018). Nanoparticles for SPECT and PET imaging: towards personalized medicine and theranostics. Curr Med Chem.

[36] Chen D, Dougherty CA, Yang D, Wu H, Hong H (2016). Radioactive nanomaterials for multimodality imaging. Tomography.

[37] Zhao L, Wen S, Shi X, Zhao J (2016). ^99m^Tc labeled multifunctional polyethylenimine-entrapped gold nanoparticles for dual mode SPECT and CT imaging. J Nucl Med.

[38] Chen L, Zhong X, Yi X, Huang M, Ning P, Liu T (2015). Radionuclide ^131^I labeled reduced graphene oxide for nuclear imaging guided combined radio- and photothermal therapy of cancer. Biomaterials.

[39] Sun G, Wang T, Li X, Li D, Peng Y, Wang X (2018). Sub-micrometer Au@ PDA-^125^I particles as theranostic embolism beads for radiosensitization and SPECT/CT monitoring. Adv Healthc Mater.

[40] Zhang R, Xiong C, Huang M, Zhou M, Huang Q, Wen X (2011). Peptide-conjugated polymeric micellar nanoparticles for dual SPECT and optical imaging of EphB4 receptors in prostate cancer xenografts. Biomaterials.

[41] Yook S, Cai Z, Lu Y, Winnik MA, Pignol J-P, Reilly RM (2016). Intratumorally injected ^177^Lu-labeled gold nanoparticles: gold nanoseed brachytherapy with application for neoadjuvant treatment of locally advanced breast cancer. J Nucl Med.

[42] Wang S, Lee RJ, Mathias CJ, Green MA, Low PS (1996). Synthesis, purification, and tumor cell uptake of ^67^Ga-deferoxamine-folate, a potential radiopharmaceutical for tumor imaging. Bioconjugate Chem.

[43] Ni D, Jiang D, Ehlerding EB, Huang P, Cai W (2018). Radiolabeling silica-based nanoparticles via coordination chemistry: basic principles, strategies, and applications. Acc Chem Res.

[44] Zhong X, Yang K, Dong Z, Yi X, Wang Y, Ge C (2015). Polydopamine as a biocompatible multifunctional nanocarrier for combined radioisotope therapy and chemotherapy of cancer. Adv Funct Mater.

[45] Song M, Liu N, He L, Liu G, Ling D, Su X (2018). Porous hollow palladium nanoplatform for imaging-guided trimodal chemo-, photothermal-, and radiotherapy. Nano Res.

[46] Kim YH, Jeon J, Hong SH, Rhim WK, Lee YS, Youn H (2011). Tumor targeting and imaging using cyclic RGD-PEGylated gold nanoparticle probes with directly conjugated iodine-125. Small.

[47] Chen M, Guo Z, Chen Q, Wei J, Li J, Shi C (2018). Pd nanosheets with their surface coordinated by radioactive iodide as a high-performance theranostic nanoagent for orthotopic hepatocellular carcinoma imaging and cancer therapy. Chem Sci.

[48] Guo Z, Chen M, Peng C, Mo S, Shi C, Fu G (2018). pH-sensitive radiolabeled and superfluorinated ultra-small palladium nanosheet as a high-performance multimodal platform for tumor theranostics. Biomaterials.

[49] Liu Y, Ai K, Lu L (2012). Nanoparticulate X-ray computed tomography contrast agents: from design validation to *in vivo* applications. Acc Chem Res.

[50] Dou Y, Guo Y, Li X, Li X, Wang S, Wang L (2016). Size-tuning ionization to optimize gold nanoparticles for simultaneous enhanced CT imaging and radiotherapy. ACS Nano.

[51] Popovtzer R, Agrawal A, Kotov NA, Popovtzer A, Balter J, Carey TE (2008). Targeted gold nanoparticles enable molecular CT imaging of cancer. Nano Lett.

[52] Wang H, Zheng L, Peng C, Shen M, Shi X, Zhang G (2013). Folic acid-modified dendrimer-entrapped gold nanoparticles as nanoprobes for targeted CT imaging of human lung adencarcinoma. Biomaterials.

[53] Ni D, Bu W, Ehlerding EB, Cai W, Shi J (2017). Engineering of inorganic nanoparticles as magnetic resonance imaging contrast agents. Chem Soc Rev.

[54] Lee N, Hyeon T (2012). Designed synthesis of uniformly sized iron oxide nanoparticles for efficient magnetic resonance imaging contrast agents. Chem Soc Rev.

[55] Mi P, Cabral H, Kokuryo D, Rafi M, Terada Y, Aoki I (2013). Gd-DTPA-loaded polymer-metal complex micelles with high relaxivity for MR cancer imaging. Biomaterials.

[56] Zhu X, Chi X, Chen J, Wang L, Wang X, Chen Z (2015). Real-time monitoring *in vivo* behaviors of theranostic nanoparticles by contrast-enhanced T1 imaging. Anal Chem.

[57] Zhao H, Zhao L, Wang Z, Xi W, Dibaba ST, Wang S (2019). Heterogeneous growth of palladium nanocrystals on upconversion nanoparticles for multimodal imaging and photothermal therapy. J Mater Chem B.

[58] Hirsch LR, Stafford RJ, Bankson JA, Sershen SR, Rivera B, Price R (2003). Nanoshell-mediated near-infrared thermal therapy of tumors under magnetic resonance guidance. Proc Natl Acad Sci USA.

[59] Xia Y, Li W, Cobley CM, Chen J, Xia X, Zhang Q (2011). Gold nanocages: from synthesis to theranostic applications. Acc Chem Res.

[60] Yang X, Yang M, Pang B, Vara M, Xia Y (2015). Gold Nanomaterials at work in biomedicine. Chem Rev.

[61] Jaque D, Martínez Maestro L, del Rosal B, Haro-Gonzalez P, Benayas A, Plaza JL (2014). Nanoparticles for photothermal therapies. Nanoscale.

[62] Kennedy WJ, Izor S, Anderson BD, Frank G, Varshney V, Ehlert GJ (2018). Thermal reshaping dynamics of gold nanorods: influence of size, shape, and local environment. ACS Appl Mater Interfaces.

[63] Tang S, Chen M, Zheng N (2014). Sub-10-nm Pd nanosheets with renal clearance for efficient near-infrared photothermal cancer therapy. Small.

[64] Shi S, Huang Y, Chen X, Weng J, Zheng N (2015). Optimization of surface coating on small Pd nanosheets for *in vivo* near-infrared photothermal therapy of tumor. ACS Appl Mater Interfaces.

[65] Wang C, Li Y, Shi X, Zhou J, Zhou L, Wei S (2018). Use of an NIR-light-responsive CO nanodonor to improve the EPR effect in photothermal cancer treatment. Chem Commun.

[66] Gao G, Jiang Y-W, Jia H-R, Sun W, Guo Y, Yu X-W (2019). From perinuclear to intranuclear localization: a cell-penetrating peptide modification strategy to modulate cancer cell migration under mild laser irradiation and improve photothermal therapeutic performance. Biomaterials.

[67] Li X, Lee S, Yoon J (2018). Supramolecular photosensitizers rejuvenate photodynamic therapy. Chem Soc Rev.

[68] Zhu H, Li J, Qi X, Chen P, Pu K (2018). Oxygenic hybrid semiconducting nanoparticles for enhanced photodynamic therapy. Nano Lett.

[69] Kim J, Cho HR, Jeon H, Kim D, Song C, Lee N (2017). Continuous O_2_-evolving MnFe_2_O_4_ nanoparticle-anchored mesoporous silica nanoparticles for efficient photodynamic therapy in hypoxic cancer. J Am Chem Soc.

[70] Shi S, Zhu X, Zhao Z, Fang W, Chen M, Huang Y (2013). Photothermally enhanced photodynamic therapy based on mesoporous Pd@Ag@mSiO_2_ nanocarriers. J Mater Chem B.

[71] Zhao Z, Shi S, Huang Y, Tang S, Chen X (2014). Simultaneous photodynamic and photothermal therapy using photosensitizer-functionalized Pd nanosheets by single continuous wave laser. ACS Appl Mater Interfaces.

[72] Li S, Gu K, Wang H, Xu B, Li H, Shi X (2020). Degradable holey palladium nanosheets with highly active 1D nanoholes for synergetic phototherapy of hypoxic tumors. J Am Chem Soc.

[73] Sun D, Huang Y, Zhang X, Peng J, Li J, Ming J (2018). A Pd corolla-human serum albumin-indocyanine green nanocomposite for photothermal/photodynamic combination therapy of cancer. J Mater Chem B.

[74] Nam J, Son S, Ochyl LJ, Kuai R, Schwendeman A, Moon JJ (2018). Chemo-photothermal therapy combination elicits anti-tumor immunity against advanced metastatic cancer. Nat Commun.

[75] Gil Y-G, Kang S, Chae A, Kim Y-K, Min D-H, Jang H (2018). Synthesis of porous Pd nanoparticles by therapeutic chaga extract for highly efficient tri-modal cancer treatment. Nanoscale.

[76] Tang S, Chen M, Zheng N (2015). Multifunctional ultrasmall Pd nanosheets for enhanced near-infrared photothermal therapy and chemotherapy of cancer. Nano Res.

[77] Shi S, Chen X, Wei J, Huang Y, Weng J, Zheng N (2016). Platinum(IV) prodrug conjugated Pd@Au nanoplates for chemotherapy and photothermal therapy. Nanoscale.

[78] Fang W, Tang S, Liu P, Fang X, Gong J, Zheng N (2012). Pd nanosheet-covered hollow mesoporous silica nanoparticles as a platform for the chemo-photothermal treatment of cancer cells. Small.

[79] Fang W, Yang J, Gong J, Zheng N (2012). Photo- and pH-triggered release of anticancer drugs from mesoporous silica-coated Pd@Ag nanoparticles. Adv Funct Mater.

[80] Yang X, Li LL, He DG, Hai L, Tang JL, Li HF (2017). A metal-organic framework based nanocomposite with co-encapsulation of Pd@Au nanoparticles and doxorubicin for pH- and NIR-triggered synergistic chemo-photothermal treatment of cancer cells. J Mater Chem B.

[81] Zhu W, Chen M, Liu Y, Tian Y, Song Z, Song G (2019). A dual factor activated metal-organic framework hybrid nanoplatform for photoacoustic imaging and synergetic photo-chemotherapy. Nanoscale.

[82] Jiang Y-W, Gao G, Hu P, Liu J-B, Guo Y, Zhang X (2020). Palladium nanosheet-knotted injectable hydrogels formed via palladium-sulfur bonding for synergistic chemo-photothermal therapy. Nanoscale.

[83] Chen X, Zhu X, Xu T, Xu M, Wen Y, Liu Y (2019). Targeted hexagonal Pd nanosheet combination therapy for rheumatoid arthritis via the photothermal controlled release of MTX. J Mater Chem B.

[84] Wang X, Guo Z, Zhang C, Zhu S, Li L, Gu Z Ultrasmall BiOI quantum dots with efficient renal clearance for enhanced radiotherapy of cancer. Adv Sci. 2020: 1902561.

[85] Haume K, Rosa S, Grellet S, Śmiałek MA, Butterworth KT, Solov'yov AV (2016). Gold nanoparticles for cancer radiotherapy: a review. Cancer Nanotechnol.

[86] Li Y, Yun K-H, Lee H, Goh S-H, Suh Y-G, Choi Y (2019). Porous platinum nanoparticles as a high-Z and oxygen generating nanozyme for enhanced radiotherapy *in vivo*. Biomaterials.

[87] Zhang C, Yan L, Gu Z, Zhao Y (2019). Strategies based on metal-based nanoparticles for hypoxic-tumor radiotherapy. Chem Sci.

[88] Miliotou AN, Papadopoulou LC (2018). CAR T-cell therapy: a new era in cancer immunotherapy. Curr Pharm Biotechnol.

[89] Mellman I, Coukos G, Dranoff G (2011). Cancer immunotherapy comes of age. Nature.

[90] Nam J, Son S, Park KS, Zou W, Shea LD, Moon JJ (2019). Cancer nanomedicine for combination cancer immunotherapy. Nat Rev Mater.

[91] Ribas A, Wolchok JD (2018). Cancer immunotherapy using checkpoint blockade. Science.

[92] Zhu G, Zhang F, Ni Q, Niu G, Chen X (2017). Efficient nanovaccine delivery in cancer immunotherapy. ACS Nano.

[93] Chen Q, Xu L, Liang C, Wang C, Peng R, Liu Z (2016). Photothermal therapy with immune-adjuvant nanoparticles together with checkpoint blockade for effective cancer immunotherapy. Nat Commun.

[94] Hess KL, Medintz IL, Jewell CM (2019). Designing inorganic nanomaterials for vaccines and immunotherapies. Nano Today.

[95] Li Y, He L, Dong H, Liu Y, Wang K, Li A (2018). Fever-inspired immunotherapy based on photothermal CpG nanotherapeutics: the critical role of mild heat in regulating tumor microenvironment. Adv Sci.

[96] Dong X, Liang J, Yang A, Qian Z, Kong D, Lv F (2019). Fluorescence imaging guided CpG nanoparticles-loaded IR820-hydrogel for synergistic photothermal immunotherapy. Biomaterials.

[97] Zhang H, Cheng T, Lai L, Deng S, Yu R, Qiu L (2018). BN nanospheres functionalized with mesoporous silica for enhancing CpG oligodeoxynucleotide-mediated cancer immunotherapy. Nanoscale.

[98] Ming J, Zhang J, Shi Y, Yang W, Li J, Sun D (2020). A trustworthy CpG nanoplatform for highly safe and efficient cancer photothermal combined immunotherapy. Nanoscale.

[99] Ohsawa I, Ishikawa M, Takahashi K, Watanabe M, Nishimaki K, Yamagata K (2007). Hydrogen acts as a therapeutic antioxidant by selectively reducing cytotoxic oxygen radicals. Nat Med.

[100] Dole M, Wilson FR, Fife WP (1975). Hyperbaric hydrogen therapy: a possible treatment for cancer. Science.

[101] Chen L, Zhou SF, Su L, Song J (2019). Gas-mediated cancer bioimaging and therapy. ACS Nano.

[102] Wu Y, Yuan M, Song J, Chen X, Yang H (2019). Hydrogen gas from inflammation treatment to cancer therapy. ACS Nano.

[103] Li S, Liao R, Sheng X, Luo X, Zhang X, Wen X (2019). Hydrogen gas in cancer treatment. Front Oncol.

[104] Zhao Z, Huang X, Li M, Wang G, Lee C, Zhu E (2015). Synthesis of stable shape-controlled catalytically active beta-palladium hydride. J Am Chem Soc.

[105] Dai Y, Mu X, Tan Y, Lin K, Yang Z, Zheng N (2012). Carbon monoxide-assisted aynthesis of aingle-crystalline Pd tetrapod nanocrystals through hydride formation. J Am Chem Soc.

[106] Zhou G, Wang YS, Jin Z, Zhao P, Zhang H, Wen Y (2019). Porphyrin-palladium hydride MOF nanoparticles for tumor-targeting photoacoustic imaging-guided hydrogenothermal cancer therapy. Nanoscale Horiz.

[107] Wang Y, Biby A, Xi Z, Liu B, Rao Q, Xia X (2019). One-pot synthesis of single-crystal palladium nanoparticles with controllable sizes for applications in catalysis and biomedicine. ACS Appl. Nano Mater.

[108] Chankeshwara SV, Indrigo E, Bradley M (2014). Palladium-mediated chemistry in living cells. Curr Opin Chem Biol.

[109] Miller MA, Askevold B, Mikula H, Kohler RH, Pirovich D, Weissleder R (2017). Nano-palladium is a cellular catalyst for *in vivo* chemistry. Nat Commun.

[110] Santra M, Ko S-K, Shin I, Ahn KH (2010). Fluorescent detection of palladium species with an O-propargylated fluorescein. Chem Commun.

[111] Spicer CD, Triemer T, Davis BG (2012). Palladium-mediated cell-surface labeling. J Am Chem Soc.

[112] Wang F, Zhang Y, Du Z, Ren J, Qu X (2018). Designed heterogeneous palladium catalysts for reversible light-controlled bioorthogonal catalysis in living cells. Nat Commun.

[113] Sancho-Albero M, Rubio-Ruiz B, Pérez-López AM, Sebastián V, Martín-Duque P, Arruebo M (2019). Cancer-derived exosomes loaded with ultrathin palladium nanosheets for targeted bioorthogonal catalysis. Nat Catal.

[114] Weiss JT, Dawson JC, Macleod KG, Rybski W, Fraser C, Torres-Sanchez C (2014). Extracellular palladium-catalysed dealkylation of 5-fluoro-1-propargyl-uracil as a bioorthogonally activated prodrug approach. Nat Commun.

[115] Hoop M, Ribeiro AS, Rösch D, Weinand P, Mendes N, Mushtaq F (2018). Mobile magnetic nanocatalysts for bioorthogonal targeted cancer therapy. Adv Funct Mater.

[116] Adam C, Pérez-López AM, Hamilton L, Rubio-Ruiz B, Bray TL, Sieger D (2018). Bioorthogonal uncaging of the active metabolite of irinotecan by palladium-functionalized microdevices. Chem-Eur J.

[117] Indrigo E, Clavadetscher J, Chankeshwara SV, Lilienkampf A, Bradley M (2016). Palladium-mediated *in situ* synthesis of an anticancer agent. Chem Commun.

[118] Chen X, Li J, Huang Y, Wei J, Sun D, Zheng N (2017). The biodistribution, excretion and potential toxicity of different-sized Pd nanosheets in mice following oral and intraperitoneal administration. Biomater Sci.

[119] Huang Y, Chen X, Shi S, Chen M, Tang S, Mo S (2015). Effect of glutathione on *in vivo* biodistribution and clearance of surface-modified small Pd nanosheets. Sci China-Chem.

[120] Li J, Liu H, Ming J, Sun D, Chen X, Liu X (2019). The biobehavior, biocompatibility and theranostic application of SPNS and Pd@Au nanoplates in rats and rabbits. Chem Sci.

[121] Zhang L, Guo S, Dong S, Wang E (2012). Pd nanowires as new biosensing materials for magnified fluorescent detection of nucleic acid. Anal Chem.

[122] Wang Y, Chen M, Wang S, Du S, Zheng X, Jiang X (2018). Size-tunable two-dimensional Pd@Au nanoplates as a platform for fluorescence sensing. J Chin Chem Soc.

[123] Wei J, Chen X, Shi S, Mo S, Zheng N (2015). An investigation of the mimetic enzyme activity of two-dimensional Pd-based nanostructures. Nanoscale.

[124] Fang G, Li W, Shen X, Perez-Aguilar JM, Chong Y, Gao X (2018). Differential Pd-nanocrystal facets demonstrate distinct antibacterial activity against Gram-positive and Gram-negative bacteria. Nat commun.

[125] Sun D, Pang X, Cheng Y, Ming J, Xiang S, Zhang C (2020). Ultrasound-switchable nanozyme augments sonodynamic therapy against multidrug-resistant bacterial infection. ACS Nano.

